# Life expectancy and causes of death in classical laminopathic progeroid syndromes: systematic review with individual-patient data synthesis

**DOI:** 10.18632/aging.206389

**Published:** 2026-06-18

**Authors:** Carlos López-Vila, Manuel García-Cordeiro, Luís Estévez-Martínez, Everardo J. Díaz-López, Teresa Prado-Moraña, Antía Fernández-Pombo, Ana I. Castro, Sofia Sánchez-Iglesias, David Araújo-Vilar

**Affiliations:** 1 UETeM-Molecular Pathology Group, Department of Psychiatry, Radiology, Public Health, Nursing and Medicine, IDIS-CIMUS, University of Santiago de Compostela, 15706 Santiago de Compostela, Spain; 2 Division of Endocrinology and Nutrition, University Clinical Hospital of Santiago de Compostela, 15706 Santiago de Compostela, Spain; 3 CIBER Fisiopatología de la Obesidad y la Nutrición (CIBERobn), 28029 Madrid, Spain

**Keywords:** progeria, Lamin A/C *(LMNA)*, *ZMPSTE24*, survival, cause of death

## Abstract

Classical laminopathic progeroid syndromes link nuclear-envelope defects to accelerated aging, but the specific prognosis for each subtype remains unclear. We conducted a PRISMA-guided systematic review and individual-patient data (IPD) synthesis (PROSPERO CRD420251080312; PubMed/Scopus to 16 July 2025). Genetically confirmed IPD constituted the primary cohort; causes of death were harmonized a priori and survival assessed by Kaplan–Meier with log-rank tests. We included 169 studies and two institutional cases, previously illustrated in the literature but not comprehensively characterized, yielding 158 genetically confirmed IPD (61 deaths, 97 censored). Median survival (years, 95% CI) was: HGPS 16.0 (13.42–19.0; n=60), MAD-B 37.0 (24–44; n=21), RD-LMNA 0.92 (0.096–2.50; n=8), and RD-ZMPSTE24 0.03 (0.014–0.047; n=38); MAD-A had no deaths (n=31). Cause-of-death profiles were subtype-specific: respiratory failure predominated in RD (36/45 deaths), cardiovascular causes in HGPS (6/11), and renal complications in MAD-B (4/5). Findings were robust in sensitivity analyses (including clinical cases, risk-of-bias exclusions, and center-specific checks). In sum, survival and mortality patterns differ markedly across classical laminopathic progeria; genetically confirmed IPD resolved by subtype provides more reliable estimates for clinical counseling and trial design in ultra-rare progeroid disorders.

## INTRODUCTION

Laminopathic progeroid syndromes provide an interesting line of research into human aging by linking defined molecular lesions with accelerated clinical trajectories. Among these conditions, there is a group that we define for this study as classical laminopathic progeria, which consists of Hutchinson-Gilford progeria (HGPS), mandibuloacral dysplasia types A and B (MAD-A/MAD-B), and restrictive dermopathy (RD). These conditions arise from pathogenic variants in the *LMNA* gene or in the prelamin-A processing protease ZMPSTE24, thereby disrupting lamin A/C, compromising nuclear architecture and genome stability [1]. For clarity, we exclude other laminopathies and non-lamin A/C progeroid disorders (e.g., *LMNA*-associated atypical progeroid syndromes, type 2 familial partial lipodystrophy, *BANF1*-associated Nestor-Guillermo progeria) from the “classical” definition used here.

HGPS (first described by Jonathan Hutchinson [2]) is classically caused by the *de novo LMNA* c.1824C>T (p.G608G) variant, and less commonly by c.1822G>A (p.G608S) variant [3, 4]. The former is a synonymous change, the latter a missense change; both create an exonic cryptic donor splice site that yields a protein called progerin [5]. MAD-A results from *LMNA* variants, whereas MAD-B is caused by *ZMPSTE24* deficiency [6, 7]. RD, meanwhile, is predominantly associated with biallelic *ZMPSTE24* variants [8] or, more rarely, with *LMNA* variants [9]. Despite shared nuclear-envelope pathology, these conditions differ markedly in tempo and organ involvement, suggesting subtype-specific survival and cause-of-death profiles rather than a uniform “progeroid” course.

For HGPS, natural-history cohorts place median survival in the mid-teens (~14.6 years) [10, 11], slightly variable in treated patients [12]. In RD, lethality clusters in the neonatal period [13]. In contrast, evidence for MAD-A/MAD-B and for RD-LMNA versus RD-ZMPSTE24 remains fragmented, often limited to case reports. Prior literature frequently mixed clinically labeled cases with genetically confirmed cases or reported pooled aggregates, impeding comparable estimation across subtypes [10, 14] and hindering subtype-specific prognostic counseling and trial planning.

To our knowledge, there is no integrated synthesis that (i) compares survival between subtypes using individual-patient data (IPD), (ii) harmonizes causes of death, and (iii) evaluates how diagnostic status (genetic vs. clinical) influences age and survival distributions. To address these gaps, we conducted a systematic review with IPD synthesis of classical laminopathic progeria (PROSPERO CRD420251080312), following PRISMA. We prespecified genetically confirmed IPD as the primary analytic cohort to minimize misclassification, used standardized cause-of-death categories, and performed sensitivity analyses that included clinically diagnosed cases. We also contributed two genetically confirmed cases previously illustrated in the literature but not comprehensively characterized or integrated into individual-patient data analyses. Our primary objectives were to estimate survival by subtype and describe the distributions of causes of death; secondary objectives evaluated the impact of diagnostic status (genetic vs. clinical) on these outcomes.

Insights from these rare syndromes help distinguish disease-accelerated from physiological aging and may guide the development of targeted interventions and clinically meaningful endpoints.

## MATERIALS AND METHODS

### Study design and registration

This systematic review followed PRISMA 2020 and was prospectively registered in PROSPERO (CRD420251080312). The research question and PICOS eligibility criteria were defined a priori, as we show in Supplementary Table 1. The PRISMA flow diagram is shown in Figure 1. The PRISMA 2020 checklist is reported in Supplementary File 2, and protocol deviations are provided in Supplementary File 1.

**Figure 1:**
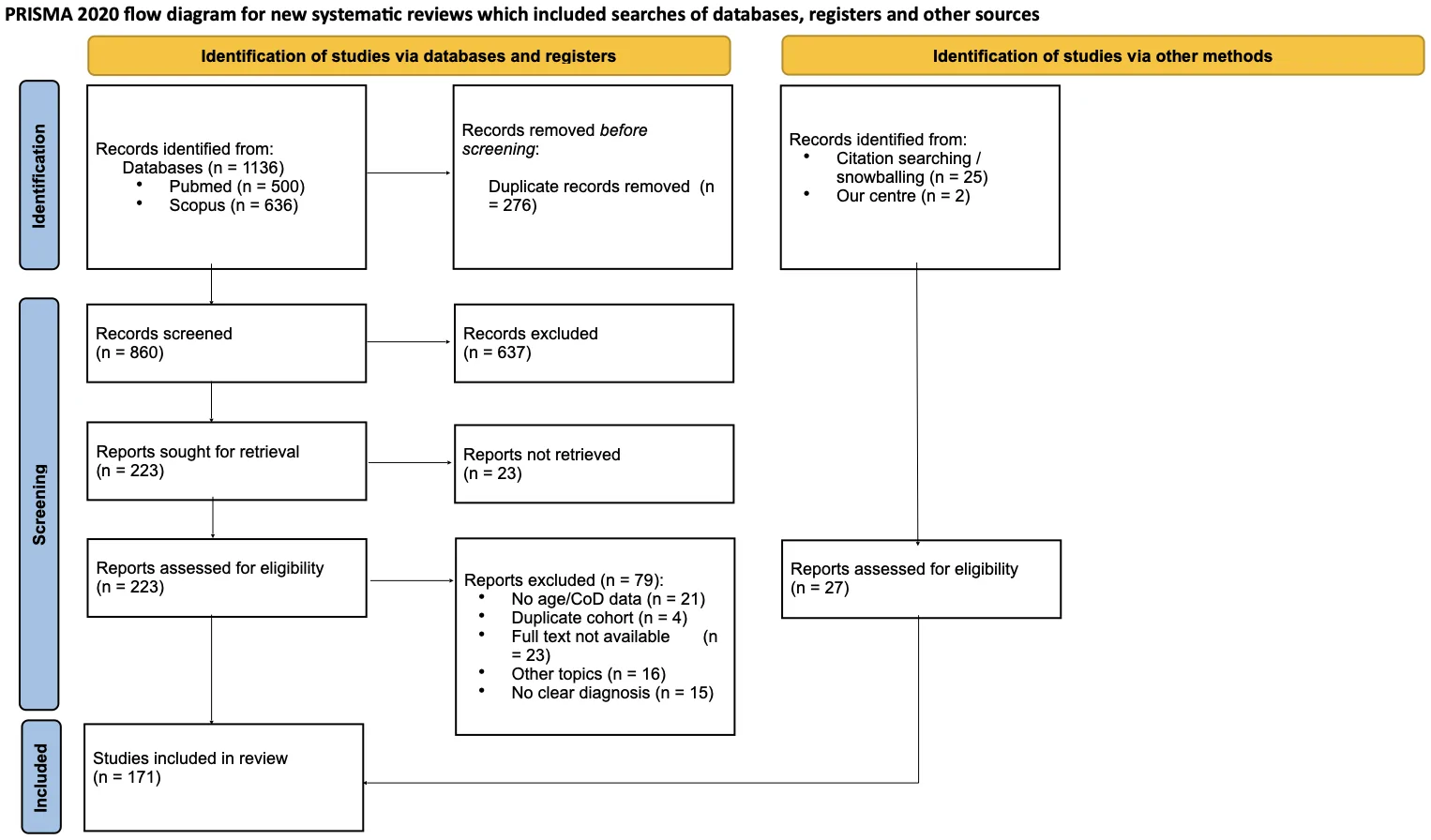
**PRISMA 2020 flow diagram of study identification, screening, eligibility, and inclusion.** Records were identified in PubMed and Scopus (from inception to 16 July 2025) and through citation chasing. After duplicate removal (n = [276]), n = [860] records were screened; n = [223] reports were sought for retrieval and n = [23] were not retrieved. n = [200] reports from databases and n = [27] from other methods were assessed for eligibility. n = [79] reports were excluded with reasons. n = [171] studies were included in the review. Exact counts by node and reasons for exclusion are detailed in Supplementary File 7; PRISMA 2020 checklist in Supplementary File 2.

### Eligibility criteria (PICOS)

### Population

Individuals of any age and sex with classical laminopathic progeroid syndromes: HGPS limited to *LMNA* c.1824C>T, p.(G608G) and c.1822G>A, p.(G608S); MAD-A due to *LMNA* variants; MAD-B due to *ZMPSTE24* variants; RD due to *ZMPSTE24* (RD-*ZMPSTE24*) or *LMNA* (RD-*LMNA*) variants.

### Diagnosis

Accepted if (i) genetically confirmed by pathogenic or likely pathogenic *LMNA*/*ZMPSTE24* variants, according to ACMG/AMP criteria, identified by Sanger, targeted NGS panel, exome, or equivalent; or (ii) clinical criteria clearly described and phenotypically compatible with the corresponding subtype.

### Study designs included

Case reports and case series with extractable data and observational clinical studies (retrospective/prospective, including comparative cohorts).

### Minimum outcomes required

Age at death; if death was not reported (or patient alive), age at last follow-up for censoring.

### Information sources and search strategy

We searched PubMed and Scopus from inception to 16 July 2025, with no date restrictions. EMBASE and Web of Science were not searched due to institutional license constraints. Given the extreme rarity of classical laminopathic progeroid syndromes and our focus on individual patient data, we anticipated a low incremental yield beyond Scopus, which indexes a broad set of biomedical journals with substantial overlap with WoS and partial overlap with EMBASE. To mitigate database limitations, we performed structured citation chasing (backward and forward) for all included records and relevant reviews.

Search terms included “Hutchinson–Gilford progeria syndrome”, HGPS, “mandibuloacral dysplasia”, “restrictive dermopathy”, LMNA, and ZMPSTE24, among others. Filters were applied to restrict records to human studies, six languages (English, Spanish, Portuguese, Italian, French, German), and study designs expected to report patient-level data (case reports and case series that present extractable clinical data, clinical/observational/comparative studies, retrospective or prospective cohorts). Review articles (narrative/systematic) and meta-analyses were used for citation chasing (snowballing) to identify additional primary reports, but they were not eligible for quantitative analyses. Full, database-specific search strategies (syntax and limits) are reported in Supplementary File 1.

### Study selection

As prespecified in PROSPERO (CRD420251080312), two reviewers independently screened titles/abstracts and full texts. Inter-rater agreement was quantified with Cohen’s κ on an initial 10% calibration subset (κ = 0.88; 95% CI 0.65–1.00) and then reassessed at full-text on the entire dataset (κ = 0.90; 95% CI 0.84–0.96). Disagreements were resolved by consensus or a third reviewer.

Reasons for full-text exclusion were recorded (Supplementary File 6), and the identification/selection process is summarized in the PRISMA flow diagram. The full screening checklist and the exclusion taxonomy are provided in Supplementary Methods (Supplementary File 1) and in coding rules (Supplementary File 7).

### Data extraction

Two reviewers independently extracted data using a piloted, structured Excel template. We collected: bibliographic data (PMID, author, year), number of patients, clinical subtype (HGPS, MAD-A, MAD-B, RD-*LMNA*, RD-*ZMPSTE24*), diagnostic method (genetically confirmed vs. clinical-only), genotype (when available), age at last follow-up or death, death status (yes/no), and cause of death (categorized as cardiovascular failure, ischemic stroke, trauma/accident, respiratory failure, sepsis, other, or not available). Discrepancies were resolved by consensus/third reviewer. To avoid double counting, potential overlaps were checked (center/period/variant/phenotype) and the most informative report was retained; a source flag (published vs. institutional) was maintained for sensitivity analyses. Primary analyses were based exclusively on genetically confirmed individual patient data (IPD) with reported age. Studies with pooled data were excluded from the main analyses; their results are presented separately as study-level context. Two genetically confirmed HGPS cases from our center were pseudonymized and integrated into the dataset. These patients were previously illustrated in the literature [15–20] in a fragmented manner but not comprehensively described or integrated into IPD analyses.

### Risk of bias/quality assessment

Methodological quality was appraised with tools matched to study design: the JBI checklists for case reports and case series, and the Newcastle–Ottawa Scale (NOS) for observational comparative/cohort studies. Two reviewers assessed quality independently; disagreements were resolved by consensus or by a third reviewer. Quality ratings did not determine inclusion, but informed sensitivity analyses.

### Outcomes and definitions

The primary outcome was overall survival, estimated as median survival using Kaplan-Meier analysis (time scale: age from birth). The co-primary outcome was the distribution of causes of death into prespecified, mutually exclusive categories: cardiovascular failure, ischemic stroke, trauma/accident, respiratory failure, sepsis, other, and not available (n.a.). Detailed coding rules and examples are provided in Supplementary File 7.

Secondary analyses compared these outcomes between genetically confirmed and clinical-only diagnoses and across subtypes, and incorporated two genetically confirmed institutional cases (source-flagged) into sensitivity analyses.

### Statistical analysis

IPD were extracted whenever available and used for survival analyses; studies reporting only aggregate values (e.g., mean/median age at death) were excluded from Kaplan-Meier analyses. As previously specified, we first compared genetically confirmed vs. clinical-only diagnoses using parametric or non-parametric tests as appropriate for continuous outcomes and χ^2^/Fisher for proportions; results informed the pooling decision prespecified in PROSPERO (CRD420251080312). Full testing details are provided in Supplementary Methods (Supplementary File 1).

Continuous summaries (age at death) are presented as mean (SD), median [IQR], and range by subtype (HGPS, MAD-A, MAD-B, RD-LMNA and RD-ZMPSTE24). Comparisons between groups for continuous outcomes were performed using the unpaired t-test/one-way ANOVA (≥3 groups) when distributions were normal; otherwise, the Mann-Whitney U test/Kruskal-Wallis test was used. For cause-of-death categories, χ^2^ tests (or Fisher's exact test when expected counts were <5) were used.

Overall survival (OS) was estimated using Kaplan-Meier analysis in genetically confirmed cases of HGPS, MAD-A, MAD-B, RD-*LMNA*, RD-*ZMPSTE24*. The time scale was age (years) from birth to the event. For records with age at death reported as 0 years, we assigned 1/365 years (~1 day) to satisfy model requirements. In the genetically confirmed IPD cohort (primary analysis), no records had age at death equal to 0 years. Three clinically diagnosed cases were recorded as 0 years and were adjusted accordingly. Given the very small number of such events, this adjustment did not materially affect the survival estimates. The 95% CIs were obtained with the Greenwood estimator, and curves were compared using the log-rank test. Studies that only reported aggregate measures -for example, mean age at death and percentages of causes of death- without individual data were excluded from KM analyses. Differences between subtypes were assessed with a global log-rank test and pairwise log-rank with Holm adjustment; two-sided α = 0.05. No formal meta-analysis of means was planned, as the primary objective was based on pooled individual data on time to event and survival curves, rather than on study-level aggregation.

We estimated cause-specific cumulative incidence functions (CIF) by subtype using the Aalen–Johansen approach; curves were shown only when event counts were adequate. Fine–Gray regression was not run due to the limited number of cause-specific events in several strata; full outputs are in the Supplement Code (Supplementary File 5).

Sensitivity analyses excluded (i) clinically diagnosed cases, (ii) studies with a high risk of bias, and (iii) the two institutional cases from our center (and reciprocal inclusion), and the key comparisons were redone.

The analyses were performed in Python with lifelines, pandas, numpy, and matplotlib (exact versions in Supplementary File 5).

### Institutional cases and ethics

In addition to records identified in our systematic search, we included two genetically confirmed cases of classic Hutchinson–Gilford progeria syndrome managed at Complexo Hospitalario Universitario de Santiago de Compostela (Spain), which had previously been reported in a fragmented manner across the literature, mainly as illustrative images or partial descriptions [15–20]. Clinical, imaging, and molecular data were abstracted from routine records; no recontact or additional procedures were undertaken.

### Data/code availability

Anonymized IPD and the codebook are provided as Supplementary Data Supplementary File 3 and Supplementary File 4, respectively. Analysis code and a computational environment file will be deposited in a public repository upon publication; a static snapshot is included as Supplementary File 5.

## RESULTS

### Case presentations

### Case 1

A boy diagnosed at birth with HGPS due to the *LMNA* c.1822G>A, p.(G608S) variant. Phenotypic features included generalized lipodystrophy, alopecia, leukomelanodermic macules, prominent cranial venous tree, bulging eyes secondary to retro-orbital fat loss, sharp nose, small normally implanted ears, micrognathia, nasal voice, dental malposition/absence, and nail dysplasia. Other findings included reducible umbilical hernia, bilateral coxa valga, and marked cutaneous sclerosis with joint contractures. He showed severe growth failure (weight persistently around 7–8 kg and height <90 cm in late childhood), with preserved cognitive development and good social interaction. Reported comorbidities included recurrent respiratory infections, probable bronchial asthma, primary hypothyroidism, myopia, hypoacusia and mild asymmetric septal hypertrophic cardiomyopathy on transthoracic echocardiography.

At 5 years of age, he was hospitalised for an occlusive dissection of the left internal carotid artery secondary to an aneurysmal cerebral lesion, resulting in an ischemic stroke with right hemiparesis and subsequent near-complete neurological recovery.

From early school age he was treated with lonafarnib (up to 75 mg in the morning and 50 mg in the evening), with good clinical tolerance apart from transient gastrointestinal symptoms and mild biochemical abnormalities. At 7 years, he sustained a fall from standing height with an occipital head impact and was admitted with a right epidural haematoma with uncal and subfalcine herniation; despite hyperosmolar therapy and rapid-sequence orotracheal intubation, he developed pulmonary haemorrhage through the endotracheal tube, haemodynamic collapse and refractory cardiorespiratory arrest, dying later the same day. See Supplementary Figure 6.

This case has been previously illustrated in the literature, with limited clinical detail and without integration into survival analyses [16, 17].

### Case 2

A 16-year-old girl with HGPS carrying the *LMNA* c.1824C>T, p.(G608G) variant was diagnosed at birth. Phenotypically, she presented with short stature, generalized lipodystrophy, cutaneous sclerosis and leukomelanodermic papulae, generalized alopecia, a small and sharp nose, nasal voice, small mouth, micrognathia, and dental crowding. Physical examination revealed dysplastic nails, joint stiffness with spontaneous dislocations of the right shoulder, coxa valga, and normal clavicles. Cardiac evaluation showed mild aortic regurgitation; echocardiography documented situs solitus, atrioventricular and ventriculoarterial concordance, and a left ventricle of normal dimensions with preserved systolic function. Gynecological assessment identified a right ovarian cyst measuring 8 × 3.5 cm and oligomenorrhea, with pubarche at 14 years of age. Abdominal ultrasound and laboratory workup disclosed hepatic steatosis and metabolic steatohepatitis, with HbA1c 5.6%, C-peptide 12.6 ng/dL, and marked hyperinsulinemia (176 mU/L). At age 16, she developed an acute myocardial infarction and died. See Supplementary Figures 7–9.

This case has been previously illustrated in the literature, with limited clinical detail and without integration into survival analyses [15, 16, 18–20].

### Results of systematic review

### Results of the screening process

The search identified 1,136 records. After duplicate removal (n = 276), 860 records were screened by title/abstract; 637 were excluded (basic/non-human research, n = 275; wrong topic, n = 263; no clinical outcomes, n = 94; no abstract, n = 5). Of 223 full texts, 79 were excluded (no age/age-at-death/cause-of-death, n = 21; duplicate cohorts, n = 4; full text unavailable, n = 23; unclear diagnosis, n = 15; other topics, n = 16). A supplementary table (Supplementary File 8) details the specific reasons for exclusion among studies classified as “unclear diagnosis.” A total of 171 studies were included: 144 from the original search, 25 from the citation chasing, and 2 cases from our center (PRISMA, Figure 1).

Across the cohort of patients (n=294), many of the reported cases were classified solely by clinical diagnosis (n=136) rather than genetic confirmation (n=158). Since age distributions were generally non-normal, we report medians (IQR) and Mann–Whitney U p-values; Welch’s t-test was used only when approximate normality held (details in Supplementary File 5).

### Genetic vs. clinical diagnosis

### HGPS

Median age at last report was lower in genetically confirmed than in clinically diagnosed cases (6.0 [3.9–10.0] vs. 11.3 [6.0–14.2] years; n=60 vs. 55; p=0.002). However, age at death did not differ (mean 12.3 vs. 13.5 years; deaths 11/60 vs. 24/55; p=0.6; t-test).

### RD

Genetically diagnosed patients were older at last report (0.04 [0.01–0.12] vs. 0.01 [0.00–0.05] years; n=46 vs. 55; p=0.0006) and also died later (0.04 [0.01–0.10] vs. 0.01 [0.00–0.05] years; deaths 45/46 vs. 55/55; p=0.0009).

### MAD

Genetically confirmed cases were younger at report (12.0 [4.3–22.5] vs. 24.5 [14.3–37.8] years; n=52 vs. 26; p=0.01). The age-at-death comparison was underpowered (5 vs. 1 events). Among genetically confirmed MAD-B cases with available age at death (n=4), the median age at death was 30.5 years (IQR 18.7–38.8). Given the small number of events, these estimates should be interpreted cautiously.

In terms of the distribution of causes of death, no significant differences by diagnostic status were observed in any disease (HGPS χ^2^=4.38, *p*=0.497; RD χ^2^=4.31, *p*=0.230; MAD χ^2^=2.40, *p*=0.301). Finally, crude mortality by diagnostic status differed in HGPS (genetic 18.3% [11/60] vs. clinical 43.6% [24/55], Fisher p=0.006), but not in RD (genetic 97.8% [45/46] vs. clinical 100% [55/55], Fisher p=0.456) or MAD (genetic 9.6% [5/52] vs. clinical 3.8% [1/26], Fisher p=0.657). See Supplementary Figure 4A–4C.

### Survival (Kaplan–Meier)

In the primary cohort of genetically confirmed IPD (excluding pooled data; n = 158; 61 events, 97 censored), median overall survival was 24 years (95% CI: 14–44). The curves differed widely between subtypes (log-rank χ^2^ = 272.12; p = 1.1 × 10^–57^; Figure 2 and Table 1). Median survival years by subtype were: HGPS 16 years (95% CI: 13.4–19.0; events/censored = 11/49 n = 60), MAD-B 37 years (95% CI: 24–44; events/censored = 5/16; n = 21), RD-*LMNA* 0.92 years (95% CI: 0.10–2.50; events/censored = 7/1; n = 8) and RD-*ZMPSTE24* 0.03 years (95% CI: 0.01–0.05; events/censored = 38/0; n = 38). In the case of MAD-A (n=31), no events were reported (0 deaths/31 censored; median not reached). In pairwise comparisons (log-rank test with Holm adjustment across 10 contrasts), all subtype contrasts remained significant (family-wise α=0.05; p_Holm range 4.34×10^–30^ to 0.0024). See Supplementary Table 4.

**Table 1:** Survival by subtype in the genetically confirmed IPD cohort (primary analysis).

**Subtype**	**N**	**Events**	**Censored**	**Deaths %**	**Median survival, years**	**95% CI**
HGPS	60	11	49	18.3	16.0	13.4–19.0
MAD-A	31	0	31	0.0	NR	N.A.
MAD-B	21	5	16	23.8	37.0	24.0–44.0
RD-LMNA	8	7	1	87.5	0.92	0.10–2.50
RD-ZMPSTE24	38	38	0	100.0	0.030	0.014–0.047

KM, Kaplan-Meier; IPD, individual participant data. Values counts unless otherwise indicated. *Deaths %* is calculated over N. *Median survival* is the KM median; NR indicates the median was not reached. *95% CI* refers to Greenwood confidence intervals. Ages are expressed in years (≥1 year rounded to 1 decimal; <0.1 year to 3 decimals). This table includes only genetically confirmed cases; clinically diagnosed cases are reported in Supplementary Table 3 for sensitivity analyses.

**Figure 2:**
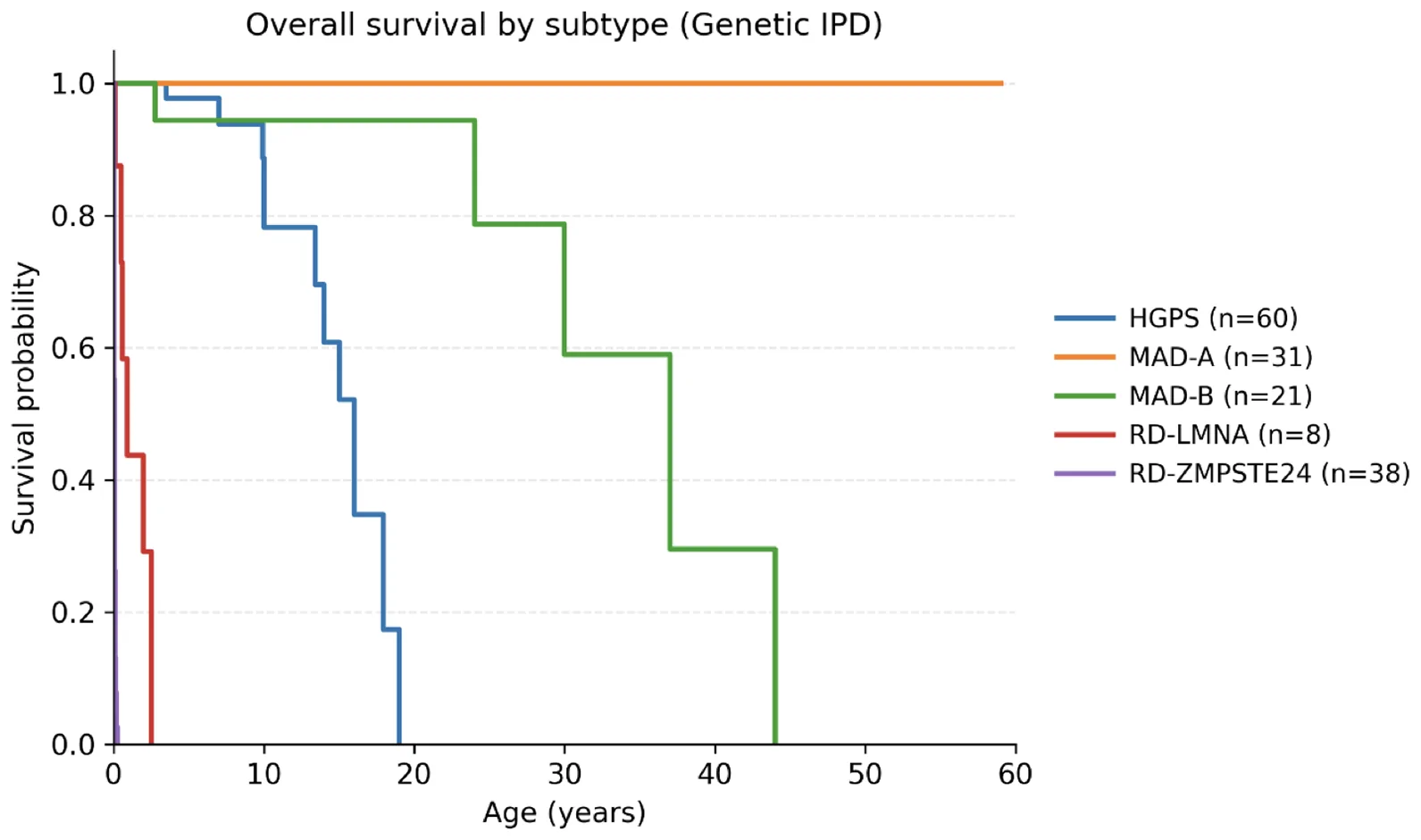
**Kaplan–Meier overall survival by subtype (genetically confirmed IPD).** Kaplan–Meier survival curves for HGPS, MAD-A, MAD-B, RD-LMNA and RD-ZMPSTE24 in the primary cohort of genetically confirmed IPD. Tick marks indicate censored observations. Numbers at risk are shown beneath the x-axis at prespecified ages. Global and pairwise log-rank tests are reported in Supplementary Table 4. A supplementary sensitivity Kaplan–Meier analysis is provided in Supplementary Figure 3.

The least marked difference, although still significant, was observed when comparing HGPS with MAD-B (p_(Holm)_ = 0.0017) and MAD-A vs. MAD-B (p_(Holm)_ = 0.0024). Tarone–Ware weighting (emphasizing early events) yielded the same pattern. In a sensitivity analysis incorporating clinical cases, trends remained unchanged: no difference for HGPS or MAD (all p > 0.05), while clinical RD showed even shorter survival than genetic RD (p = 0.0003).

### Descriptive summary (all cases)

We summarized all cases irrespective of diagnostic modality (n=294; deaths=141/294, 48%). Ages at last follow-up and at death showed a clear gradient across subtypes. Mortality was near-universal in both RD subtypes. Death was extremely early in RD-ZMPSTE24 (median age at death ≈0 years), whereas RD-LMNA showed slightly longer survival (median 0.92 years). In HGPS (n=115), ages at last follow-up and deaths clustered in adolescence (median age at death ≈12.6 y; deaths 35/115). Mandibuloacral dysplasia showed the most favorable profile: MAD-A (n=31) had no reported deaths (ages up to 59 y at last follow-up), and MAD-B (n=21) had few deaths, typically in the third decade (median age at death ≈30 years; deaths 5/21). The clinically labeled MAD group (MAD-CLIN, n=26) comprised older individuals at last report, with only one death recorded. Age and age-at-death distributions by subtype are shown in Supplementary Figures 1, 2.

### Causes of death

In the primary cohort (n=158; deaths=61; censored=97), cause-of-death profiles differed by subtype. RD was dominated by respiratory failure: 36/45 (80.0%) of RD deaths were respiratory, with RD-*LMNA* 7/7 (100%) and RD-*ZMPSTE24* 29/38 (76.3%). Unknown cause was reported in 9/45 (20.0%) RD deaths, all within RD-*ZMPSTE24*. In HGPS, the predominant cause was cardiovascular (6/11; ~55%), followed by trauma/accident (2/11; ~18%) as the second most frequent cause. In MAD-B, deaths were infrequent (5 events) and were classified under “other.” Notably, most of these “other” deaths reflected renal failure/complications (3/5; 60.0%) based on case descriptions [7, 21, 22]. MAD-A had no deaths. When multiple causes were listed, we coded the underlying primary cause according to the authors’ description.

After performing a sensitivity analysis, no differences were detected in the distribution of causes between genetic and clinical diagnoses within each disease: HGPS (Fisher 2×2 test, p = 1.00), RD (Fisher 2×2, p = 0.48), and MAD (Fisher 2×2, p = 0.33). A stacked view appears in Figure 3 and a heatmap in Supplementary Figure 5.

**Figure 3:**
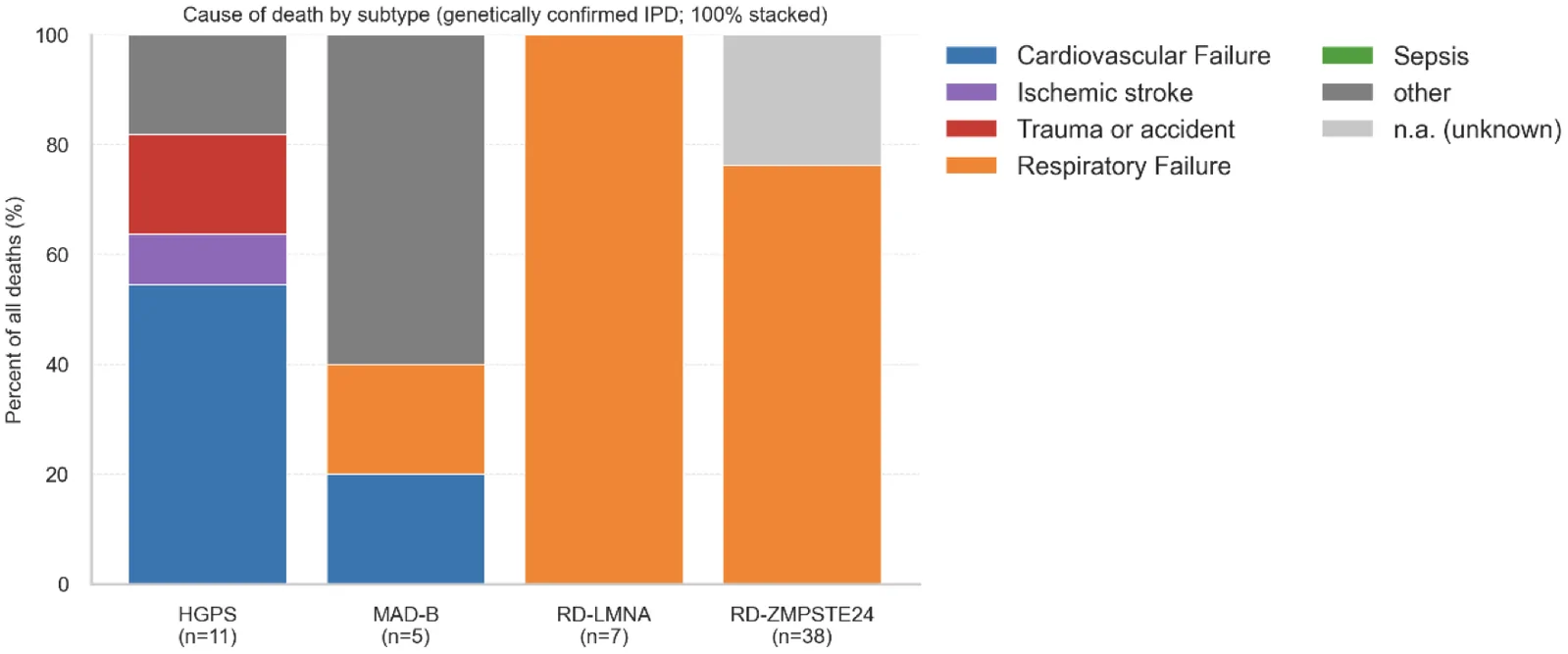
**Cause of death by subtype (Genetically confirmed IPD; 100% stacked).** Each bar shows the relative composition (100%) of all deaths by cause category within each subtype. Numbers below the x-axis indicate deaths per subtype. Categories are mutually exclusive and coded according to prespecified rules (Supplementary File 7). Exact counts and percentages are provided in Table 2.

**Table 2:** Causes of death by subtype in the genetically confirmed IPD cohort.

**Subtype**	**Deaths, n**	**Cause of death (n, %)**					
		**CV**	**Stroke**	**Trauma**	**Respiratory**	**Other**	**Unknown**
**HGPS**	11	6 (54.5%)	1 (9.1%)	2 (18.2%)	0 (0.0%)	2 (18.2%)	0 (0.0%)
**MAD-B**	5	1 (20.0%)	0 (0.0%)	0 (0.0%)	1 (20.0%)	3 (60.0%)	0 (0.0%)
**RD-LMNA**	7	0 (0.0%)	0 (0.0%)	0 (0.0%)	7 (100.0%)	0 (0.0%)	0 (0.0%)
**RD-ZMPSTE24**	38	0 (0.0%)	0 (0.0%)	0 (0.0%)	29 (76.3%)	0 (0.0%)	9 (23.7%)

Percentages are calculated over deaths within each subtype. Causes are mutually exclusive and coded according to prespecified rules (Supplementary File 7). Abbreviations: CV, cardiovascular failure; Stroke, ischemic stroke. Unknown includes not reported or ambiguous causes. Cohort includes only genetically confirmed IPD cases. Exact counts used to compute percentages are shown in the table.

### Competing risks (exploratory)

CIFs were consistent with the main analyses: cardiovascular mortality accumulates during late adolescence in HGPS, whereas respiratory/neonatal mortality rises steeply in RD; MAD-A/B contributed few cause-specific events. Causes with sparse counts (sepsis, renal) were not modeled. Details are provided in Supplementary Table 2.

### Sensitivity analysis

We tested the robustness of our findings across four prespecified scenarios.

### Including clinical IPD

Adding clinically diagnosed cases did not change the qualitative ordering of survival by subtype (see the Kaplan-Meier sensitivity analysis in Supplementary Figure 3). The distributions of causes of death followed the same trends as the main cohort. The directions of effects and inferences did not change.

### Excluding high risk of bias

Removing high-ROB studies produced only minor shifts in point estimates (median ages and proportions moved within the main-analysis CIs), with no change in statistical conclusions for between-subtype comparisons or for the cause-of-death profiles.

### Center analyses

When excluding cases from our center, estimates very similar to those of the main cohort were obtained. The “our center only” subset was, as expected, very small and lacked the statistical power to make comparisons with the main cohort.

These findings support the robustness of our main conclusions based on genetically confirmed IPD. They are detailed in Supplementary Table 3.

## DISCUSSION

In an IPD synthesis focused on genetically confirmed cases, we found a clear survival gradient between classic laminopathic progeroid syndromes (neonatal lethality in RD, median ≈16 years in HGPS, adult survival in MAD-B, and no deaths in MAD-A) and subtype-specific cause-of-death profiles (respiratory failure in RD, cardiovascular in HGPS, and renal complications in MAD-B). Genetic vs. clinical comparison showed shifts in age at last follow-up and differences in crude mortality in HGPS, with no changes in the distribution of causes. Sensitivity analyses confirmed the robustness of these conclusions.

Since the first causal genes underlying the main laminopathic progeroid syndromes were identified in 2002–2003 [3, 4], molecular confirmation has become the reference standard for diagnosis. Before that, most cases were labeled on clinical grounds alone, in a setting of overlapping phenotypes where expert adjudication was often required. This historical context plausibly introduced misclassification into the literature. We therefore contrasted genetically confirmed with clinically diagnosed cases and observed discrepancies in several age distributions across subtypes. To minimize misclassification, we prespecified genetically confirmed IPD as the primary analytic cohort, while retaining clinically diagnosed cases with unequivocal phenotypes for comparative and sensitivity analyses. Reports clearly inconsistent with current criteria for classic laminopathies were excluded; operational details are provided in the Methods.

Consistent with our a priori plan, full-text review identified multiple reports-particularly within HGPS and MAD in which the assigned label was inconsistent with current diagnostic standards. To reduce misclassification bias, we excluded studies that failed any of three pre-specified rules: (i) familial clustering in HGPS, which is expected to be sporadic (Supplementary File 8); (ii) atypical or discordant phenotypes relative to classic descriptions; and (iii) absence of genetic confirmation in otherwise doubtful cases. Illustrative examples include Sood et al. [23] and Parkash et al. [24], who describe pairs of siblings with progeroid phenotypes and prolonged survival (20, 24, and 32 years), more consistent with alternative progeroid syndromes (e.g., skeletal dysplasia or mandibuloacral dysplasia) than classic HGPS.

This conservative approach prioritizes internal validity of survival and mortality estimates. In total, n = 15 reports were excluded on these grounds.

When contrasting genetically confirmed with clinically diagnosed cases, we observed divergent patterns across subtypes. In HGPS and MAD, genetically confirmed individuals were younger at last report than clinically diagnosed cases, while the opposite pattern occurred in RD: genetically confirmed patients were older at last report and died later. Notably, in HGPS age at death did not differ between diagnostic groups, despite the shift in ages at last report. These patterns likely reflect ascertainment biases associated with the era of diagnosis (pre- vs. post-genetic confirmation) and referral pathways. Such bias may have been partially mitigated by our exclusion of reports that were clearly inconsistent with established clinical criteria or insufficiently documented.

The distribution of causes of death did not differ by diagnostic status within subtypes, indicating that the diagnostic route per se is unlikely to bias the immediate mechanisms of death captured in the literature. By contrast, crude mortality at last follow-up differed in HGPS (higher among clinically diagnosed cases) while RD and MAD showed no significant difference.

Together, these observations argue for genetically confirmed IPD as the primary analytic set when feasible, with clinical-only cases reserved for sensitivity analyses.

Our Kaplan–Meier analysis of genetically confirmed IPD shows a pronounced survival gradient across classical laminopathic progeroid syndromes, ranging from neonatal lethality in RD to adult mortality in MAD-B, with intermediate survival in HGPS and an apparently non-lethal course in MAD-A. In RD, mortality occurred very early. Most patients with the *ZMPSTE24* variants did not reach two weeks of age, while those with the *LMNA* variants survived up to eleven months. This short survival time is consistent with the known respiratory problems, chest stiffness, and skin tension that cause early respiratory failure [25–28]. In HGPS, survival declined most steeply during early-to-mid adolescence, with a median of 16 years, in line with prior reports of cardiovascular mortality in the teenage years [10, 11]. In contrast, MAD-B showed longer survival, with more than 60% of patients alive at 30 years. No deaths were documented in MAD-A, highlighting its more benign course, although the possibility of underreporting and shorter follow-up in published case reports cannot be ruled out.

Overall, these data reinforce the need for improved patient follow-up (as only few deaths have been documented in the literature, especially in MAD-A) and cautious interpretation of earlier clinical cohorts, as misclassification is likely to have biased survival estimates before the era of molecular confirmation. In addition, most published cases are isolated case reports or small series that provide only a cross-sectional “snapshot”, with little or no longitudinal follow-up, so vital status and late outcomes frequently remain undocumented.

The most robust study written to date is a large cohort published in studies led by Leslie Gordon in 2014 [11] and in 2018 [10], which concluded that median life expectancy in HGPS is around 14.5 years. The difference between our estimate of a median of 16 years for genetic HGPS and the value of 14.5 years reported by Gordon et al. [11] may be due to several factors: our stricter approach of including only genetically confirmed cases for formal analyses and our exclusion of misclassified clinical cases. This underscores the importance of distinguishing genetic vs. clinical (or aggregated) cohorts when estimating survival, as the inclusion of unconfirmed clinical cases may bias estimators toward less accurate values. This may have influenced prior survival estimates, depending on the phenotypic composition and diagnostic certainty of the included cohorts.

An additional factor that may contribute to differences across cohorts is treatment exposure. Lonafarnib, a farnesyltransferase inhibitor, was approved in 2020 following evidence of survival benefit in HGPS [12]. The large cohorts reported by Gordon et al. [10, 11] predate formal approval, whereas more recent reports reflect the therapeutic era. In our dataset, all genetically confirmed HGPS deaths derive from reports published before 2020. Among our institutional cases, only one patient was known to have received lonafarnib; this individual died at 7 years of age due to traumatic brain injury. Therefore, treatment exposure is unlikely to account for the survival estimates observed in our primary cohort. Nonetheless, as more patients are treated in the post-approval era, evolving management strategies may influence future survival comparisons.

In the genetically confirmed IPD cohort, the distribution of causes of death was subtype-specific and consistent with known pathophysiology [9, 29–32]. In RD, respiratory failure dominated, with RD-*ZMPSTE24* contributing to the majority of cases with unassigned cause (9/38). This finding is consistent with its lethal phenotype and with what is described in some narrative reviews in the literature [14, 33]. In HGPS, cardiovascular causes were the most frequent (6/11), followed by trauma/accident (2/11). Stroke-related deaths and other causes were less common: ischemic stroke accounted for 1/11 deaths, and all cases classified as “other” (2/11) corresponded to hemorrhagic stroke, resulting in a very high overall proportion of vascular etiologies (9/11, ~82%). This pattern aligns with accelerated large-vessel disease described in some studies of HGPS [31, 34, 35]. For example, in the largest cohort of HGPS [10], cardiovascular etiology was identified as the main cause, followed by trauma or accidents, with a large number of deaths without an identified cause of death. Notably, both our data and the cohort described by Gordon et al. also identifies trauma as the second most frequent cause of death, which is particularly relevant given the marked physical frailty of these patients and the potential for targeted preventive strategies to reduce trauma-related mortality.

In MAD-B, deaths were infrequent (5 total) and, when specified, were mostly renal (recorded under “other”), suggesting renal involvement as a plausible driver of mortality in this subtype and warranting focused nephrologic monitoring, a pattern also hinted at in isolated *ZMPSTE24*-related MAD case reports and reviews [7, 21, 22, 32, 36]. The prominence of renal causes in our MAD-B cohort is mechanistically plausible. In progeroid mouse models with reduced renal expression of *Zmpste24*, the accumulation of prelamin A in the kidney is associated with p53/p21 activation, TGF-β1/CTGF upregulation, interstitial fibrosis, and increased serum creatinine [37]. Similarly, MAD-B has been reported to present with proteinuric nephropathy and focal segmental glomerulosclerosis (FSGS) [22, 38]. Taken together, these observations make it plausible that the combination of various mechanisms produces segmental glomerulosclerosis, with consequent progressive renal failure documented in some patients with MAD-B [7, 21, 22].

In MAD-A, no deaths were reported and, accordingly, no causes were attributable.

Finally, a considerable share of cases lacked a reported cause of death. This was uncommon in genetically confirmed series -limited to RD-*ZMPSTE24*, where 9 of 38 deaths had no assigned cause- but it was more frequent among clinically diagnosed reports. This pattern underscores the need for standardized cause-of-death coding (e.g., ICD-aligned categories) in future registries. It also likely reflects the literature mix (older narrative summaries with mixed-certainty diagnoses), which can inflate the “unknown” category.

Our pre-specified sensitivity checks supported the robustness of the main findings. First, when we expanded the dataset to include clinically diagnosed cases, the subtype patterns for age distributions and cause-of-death profiles did not change; the only notable change reflected the descriptive results: clinical RD showed even earlier mortality than genetically confirmed RD, while HGPS and MAD remained directionally consistent. This discrepancy in RD may be due to older, clinically defined series being enriched with fulminant neonatal presentations, whereas genetically confirmed cohorts also include atypical or milder *LMNA*-related phenotypes that occasionally survive beyond the first few weeks of life. Second, excluding studies considered to be at high risk of bias did not substantially alter the point estimates or the relative order of subtypes, indicating that the main conclusions are not based on a small set of lower-quality reports. In all tests, inferences were limited when events were rare (no deaths in MAD-A; few in MAD-B), and wide intervals should be interpreted accordingly. Taken together, these analyses justify the choice of genetically confirmed individual-patient data as the primary analytic cohort while suggesting that inclusion of clinically diagnosed cases would not overturn the central conclusions.

These findings have direct clinical and research implications. Subtype-specific survival gradients and distinct mortality profiles support tailored follow-up and anticipatory counseling for families. In practice, care plans should prioritize early cardiometabolic surveillance in HGPS, structured neonatal/respiratory pathways in RD, and renal monitoring in MAD-B. For trial design, subtype-resolved, genetically confirmed IPD provide more reliable event-rate assumptions, justify stratified eligibility and endpoint selection (e.g., cause-specific composites), and inform enrollment windows (earlier in RD, broader in MAD-B). Harmonized cause-of-death coding also facilitates cross-study comparability and may help benchmark therapeutic effects (e.g., farnesyltransferase inhibition in HGPS) against clinically meaningful outcomes.

This review has limitations. First, searches were limited to PubMed and Scopus; EMBASE/Web of Science were not queried due to license constraints, so some reports may have been missed despite our structured citation chasing, which was employed to maximize coverage. Second, competing-risks results are descriptive (CIF) only and should be interpreted cautiously given small strata and the absence of Fine–Gray regression. Third, a non-trivial fraction of deaths lacked a clearly specified cause, which limits cause-resolved inference.

## CONCLUSIONS

Classical laminopathic progeria demonstrates clear subtype-specific survival and mortality profiles. Historical misclassification is common and can distort estimates if clinically labeled cases are pooled with genetically confirmed ones; analyses should prioritize genetically confirmed IPD and standardized coding of causes of death. Survival follows a subtype gradient (longest in MAD-B, intermediate in HGPS, shortest in RD), and causes of death align with disease biology (cardiovascular in HGPS, neonatal respiratory in RD, renal complications in MAD-B). The proportion of unspecified causes remains non-trivial, underscoring the need for structured follow-up and complete outcome reporting. These findings support more reliable estimates for clinical counseling, care planning, and trial design tailored to each subtype.

## Supplementary Material

Supplementary File 1

Supplementary File 2

Supplementary File 3

Supplementary File 4

Supplementary File 5

Supplementary File 6

Supplementary File 7

Supplementary File 8

Supplementary Figures

Supplementary Tables
