# Supplementary material for: Life expectancy and causes of death in classical laminopathic progeroid syndromes: systematic review with individual-patient data synthesis
Source: Aging (Albany NY). 2026 Jun 18;18(1):695–718. doi: 10.18632/aging.206389 (PMC13632853; doi:10.18632/aging.206389)
Supplement: Supplementary file 1 [file aging-18-1-206389-s001.pdf]

SD1 — Supplementary Methods

Scope. This file complements the Methods section in the main manuscript. It provides additional technical detail on: (i) information sources, (ii) full search strategies, (iii) data management and IPD handling, (iv) statistical implementations, (v) sensitivity and subgroup analyses, (vi) assessment of reporting bias and certainty of evidence, (vii) software and reproducibility, and (viii) protocol registration and deviations. Narrative already covered in the main text is not repeated.

1) Information sources:

- **Databases:** We searched PubMed (MEDLINE) and Scopus. The final searches were run on 16 July 2025.
- **Filters:** In both databases we applied filters designed to enrich for human, clinically relevant reports likely to contain extractable patient-level data:
  - Population: humans
  - Languages: English, French, German, Spanish, Portuguese, Italian
  - Study types: case reports, case series, observational or comparative cohorts, and other clinical studies where individual patient data (IPD) or minimally aggregated data could be extracted.
- **Deduplication procedure:** Records were exported to Rayyan. Deduplication used an automated matching algorithm based on combinations of title, first author, year, journal, DOI and/or PMID, followed by manual verification of uncertain pairs before final removal.
- **Additional sources:** To minimise the risk of missing eligible reports, we supplemented database searches with backward and forward citation chasing for all included records and key narrative or systematic reviews in the field. Full texts identified through citation chasing were screened with the same criteria and included when eligible.

2) Full search strategies

<u>**Database: PubMed (MEDLINE)**</u>

Search date: 16 Jul 2025

Full strategy:

(("Hutchinson-Gilford progeria syndrome"[tiab] OR "Hutchinson-Gilford"[tiab] OR HGPS[tiab] OR (progeria[tiab] AND (Hutchinson[tiab] OR Gilford[tiab] OR LMNA[tiab] OR "lamin A"[tiab] OR progerin[tiab] OR G608G[tiab]))) OR ("restrictive dermopathy"[tiab] OR "neonatal restrictive dermopathy"[tiab] OR ("restrictive dermopathy"[tiab] AND (ZMPSTE24[tiab] OR LMNA[tiab])) OR "mandibuloacral dysplasia"[tiab]))

Filters applied in PubMed:

- Species: humans
- Languages: English, French, German, Italian, Portuguese, Spanish
- Publication type: Case Reports, Clinical Study, Comparative Study, Meta-Analysis, Observational Study, Review, Systematic Review

Retrieval: 500 records

Database: Scopus

Search date: 16 Jul 2025

Strategy: ((TITLE-ABS-KEY ("Hutchinson-Gilford progeria syndrome") OR TITLE-ABS-KEY ("Hutchinson-Gilford") OR TITLE-ABS-KEY (HGPS) OR (TITLE-ABS-KEY (progeria) AND (TITLE-ABS-KEY (Hutchinson) OR TITLE-ABS-KEY (Gilford) OR TITLE-ABS-KEY (LMNA) OR TITLE-ABS-KEY ("lamin A") OR TITLE-ABS-KEY (progerin) OR TITLE-ABS-KEY (G608G)))) OR TITLE-ABS-KEY ("restrictive dermopathy") OR TITLE-ABS-KEY ("neonatal restrictive dermopathy") OR (TITLE-ABS-KEY ("restrictive dermopathy") AND (TITLE-ABS-KEY (ZMPSTE24) OR TITLE-ABS-KEY (LMNA)))) OR TITLE-ABS-KEY ("mandibuloacral dysplasia"))

Filters applied in Scopus:

- Subject area: Medicine (MEDI)
- Species: Humans
- Languages: English, French, German, Spanish, Portuguese, Italian
- Document types: Case Reports, Clinical Study, Observational Study, Comparative Study, Meta-Analysis, Systematic Review, Review

Retrieval: 636 records

Additional sources: References of included articles were manually screened (backward/forward citation chasing); full texts identified were screened with the same criteria and included when eligible.

3) Data management (exact rules)

- **Subtype normalization:** Clinical and genetic subtypes were harmonised into the following categories for analysis: HGPS, MAD-A, MAD-B, RD-LMNA, RD-ZMPSTE24. On occasion (mainly for graphs), the labels MAD-CLIN, RD-CLIN, and HGPS-CLIN were used to clarify that they belong to the subgroup with a clinical diagnosis.

- **Diagnostic status (genetic vs clinical):** Whenever the report explicitly stated that a case was genetically confirmed or clinically diagnosed, that statement was taken as the reference. In ambiguous situations, cases were conservatively classified as "clinical only" and explored in sensitivity analyses.

- **IPD versus aggregate data:** We defined IPD as a patient-level data in which each individual case could be identified (e.g., through a case ID, separate row in a table, or explicit narrative structure). Reports without distinguishable individual records were treated as aggregate. For IPD studies, each patient was extracted as a separate record. For purely aggregate reports, only summary information was used where applicable. All analyses were restricted to IPD and the primary analyses were restricted to genetically confirmed IPD, as pre-specified in the decision pathway.

- **Time scale and zero-age events:** The time scale for survival analyses was age in years from birth. When age at death was reported as 0 (i.e., death at birth), we set it to 1/365 years to allow inclusion in time-to-event analyses while preserving clinical interpretation.

- **Cause-of-death coding (mutually exclusive):** Cause of death was coded into mutually exclusive categories using a predefined dictionary (Supplementary Data SD9) based on keywords and clinical descriptions. Cause of death were mapped to one of seven categories: cardiovascular failure, ischemic stroke, trauma/accident, respiratory failure, sepsis, other specified cause, unknown/not reported.

- **Overlap handling (duplicate cohorts):** To avoid double-counting, we systematically assessed potential overlaps across reports based on centre, time-period, genotype and clinical description. When overlapping cohorts were identified:

    - We retained the most informative, IPD-rich source.
    - Discrepancies between overlapping reports were resolved by prioritising the report with clearer primary data

4) Statistical implementations

4.1 Descriptive statistics and group comparisons

- **Continuous variables** were summarised as mean (standard deviation), median [interquartile range], and range, where appropriate.

- **Normality** was assessed using the Shapiro–Wilk test.

- **Between-group comparisons** for continuous variables used:

    - t-tests or one-way ANOVA when normality and homogeneity assumptions were approximately met.

    - Mann–Whitney U or Kruskal–Wallis tests otherwise.

- **Categorical variables** were compared using χ^2 tests; Fisher's exact test was used whenever any expected cell count was < 5.

- To **control the family-wise error rate in multiple pairwise comparisons**, we applied Holm adjustment to p-values (two-sided $\alpha = 0.05$).

4.2 Survival analysis (primary cohort: genetically confirmed IPD)

- The primary time-to-event analyses were performed in the genetically confirmed IPD cohort.

- **Kaplan–Meier** curves were generated for each subtype (HGPS, MAD-A, MAD-B, RD-LMNA, RD-ZMPSTE24).

- **Median survival** and 95% confidence intervals were estimated using the Brookmeyer–Crowley method with Greenwood variance (lifelines defaults).

- We conducted both **global and pairwise log-rank tests** across subtypes. Pairwise p-values were adjusted for multiplicity using Holm's procedure.

4.3 Competing risks

- Competing-risks analyses were implemented to describe cause-specific mortality patterns.

- **Cumulative incidence functions (CIFs)** by cause and subtype were estimated using the Aalen–Johansen method.

- **Numbers at risk** were displayed at specified time points (0, 5, 10, 15, 20 years), conditional on the observed follow-up range.

- **CIF curves** were presented only when the number of events for a given cause/subtype combination was ≥ 5, to reduce unstable estimates.

- **Fine–Gray** subdistribution hazard models were not fitted because of the limited number of cause-specific events in several strata, which would have yielded unstable and potentially misleading subdistribution hazard estimates.

4.4 Cause-of-death summaries & tests

- In the main genetically confirmed IPD cohort, we summarised causes of death as counts and row-wise percentages by subtype.

- **Overall proportions** were presented with Wilson 95% confidence intervals.

- **Global differences in cause-of-death distribution by subtype** were assessed using χ^2 tests after removing all-zero rows/columns.

- For **sensitivity analyses comparing genetic versus clinical diagnoses within HGPS, MAD and RD**, rare causes were iteratively collapsed into "other" until all expected cells were ≥ 5. If the resulting table was 2×2, we reported Fisher's exact test and odds ratios with confidence intervals.

5) Sensitivity and subgroup analyses (pre-specified)

The following sensitivity and subgroup analyses were specified a priori:

1. Exclusion of **clinically diagnosed** cases, retaining only genetically confirmed IPD.

2. Exclusion of studies at **high risk of bias** according to the chosen tool.

3. Inclusion versus exclusion of the **two institutional case**s contributed by the authors.

Results from these analyses are reported in the main text and in supplementary tables and were used to assess the robustness of the primary findings.

6) Reporting bias and certainty of evidence

6.1 Assessment of reporting bias:

We did not perform a formal quantitative assessment of reporting bias (e.g., funnel plots or tests for small-study effects). This decision was based on:

- The predominance of case reports and small case series.

- The lack of a sufficient number of comparable studies per outcome/subtype to support reliable small-study effect analyses.

However, we attempted to mitigate the risk of reporting bias by:

- Using two large databases with broad search strategies.
- Supplementing electronic searches with structured citation chasing.
- Including both positive and negative outcome reports whenever IPD or extractable data were available.

Limitations related to potential reporting bias and publication bias are discussed in the main manuscript.

6.2 Certainty of evidence

Given that the evidence base is dominated by IPD from case reports and small series, we did not apply a formal certainty-of-evidence framework such as GRADE. Instead, we qualitatively appraised the strength and consistency of the evidence by considering:

- Risk of bias at the study level.
- Precision of survival and cause-of-death estimates.
- Consistency of patterns across sensitivity and subgroup analyses.
- These aspects are discussed in detail in the Discussion section.

7) Software and reproducibility

- **Programming language:** Python 3.13.9

- **Core libraries:**

  - pandas 2.2.3
  - numpy 2.2.1
  - scipy 1.14.1
  - lifelines 0.30.0
  - matplotlib 3.10.0
  - seaborn 0.13.2

- **Reproducible code and data:**

  - **SD7_Code.zip**: all analysis scripts

  - **SD5_Dataset_IPD.xlsx:** anonymised individual patient data used in the primary analyses.

  - **SD6_Codebook.xlsx:** detailed codebook describing variable names, encodings and allowable values.

All scripts are written to run end-to-end on the anonymised dataset and will be deposited in a public repository upon publication, together with environment specifications, to facilitate reproducibility.

8) Protocol registration and deviations:

The review protocol was prospectively registered in PROSPERO under the title "Life Expectancy and Causes of Death in Classical Laminopathic Progeroid Syndromes: Two New Case Reports and Systematic Review" (CRD420251080312). The record was first published on 29 June 2025, with an updated version (2.0) released on 12 November 2025 after completion of the main analyses and clarification of minor wording issues, and a final administrative update on 28 November 2025 (version 3.0).

In line with the registered protocol, we predefined:

- **Population and syndromes**: patients of any age and sex with classical laminopathic progeroid syndromes: Hutchinson–Gilford progeria syndrome (HGPS), mandibuloacral dysplasia type A (MAD-A), mandibuloacral dysplasia type B (MAD-B), and restrictive dermopathy (RD) caused by pathogenic variants in LMNA or ZMPSTE24.

- **Eligibility criteria**: inclusion of non-randomised clinical designs (case reports/series, observational or comparative cohorts) providing, at a minimum, vital status, age at death or last age if alive, and cause of death; exclusion of non-human studies, conference abstracts, narrative reviews, guidelines and overlapping or duplicate cohorts.

- **Information sources and searching:** primary searches in PubMed and Scopus without date restrictions, restricted to English, French, German, Italian, Portuguese and Spanish, plus citation chasing ("snowballing") through reference lists and citing articles of included studies and key reviews.

- **Screening and data collection processes:** independent screening and data extraction by at least two reviewers (or person/machine combination) with a process to resolve discrepancies; no contact with original authors and no IPD requested from investigators or repositories beyond published material.

- **Risk-of-bias tools:** use of the Newcastle–Ottawa Scale for non-randomised observational/cohort studies and the JBI critical appraisal checklists for case reports and case series, with independent duplicate assessments.

- **Primary and secondary outcomes:** (i) life expectancy (age from birth to death or last known age if alive) and (ii) cause of death classified a priori into cardiovascular failure, ischaemic stroke, trauma/accident, respiratory failure, sepsis, other, and unknown; plus comparisons between genetically confirmed vs clinically diagnosed cases and between subtypes (HGPS, MAD-A, MAD-B, RD), and inclusion of two new genetically confirmed cases from our centre.

- **Planned synthesis and analyses:** preferential use of individual-patient data (IPD) when available; descriptive statistics of age at death; Kaplan–Meier survival curves and log-rank tests by subtype; comparisons of age at death using non-parametric tests when appropriate; comparisons of cause-of-death distributions using χ^2 or Fisher's exact tests; and pre-specified sensitivity analyses by diagnostic method (genetic vs clinical), risk of bias and inclusion/exclusion of the two institutional cases.

- The PROSPERO record explicitly stated that **risk of bias due to missing results (reporting bias) and formal certainty-of-evidence grading (e.g. GRADE) would not be assessed**. Consistent with this, we did not perform funnel plots, small-study effect tests or GRADE tables, and instead provided a qualitative appraisal of potential reporting bias and overall evidence strength in the Discussion.

Deviations and clarifications relative to the registered protocol:

Relative to the original registration, the updated **PROSPERO record (version 2.0)** introduced the following clarifications and minor amendments: handling of mixed

cohorts was further specified; we specified that reviews and meta-analyses were limited to citation tracking only; explicit criteria for excluding duplicate and overlapping cohorts were added; the wording of the planned analyses was aligned across sections; the number of newly reported cases was corrected to two (in sections where “three” had previously been stated); the genetic notation of the two classic HGPS mutations was standardised; it was clarified that no formal assessment of risk of bias due to missing results was planned; and no additional IPD would be sought from study investigators or repositories. Other minor editorial changes were implemented without modifying the study objectives, eligibility criteria or main analysis plan.

In a subsequent administrative update (**PROSPERO version 3.0**), the registered title was refined from “Life Expectancy and Causes of Death in Classical Laminopathic Progeroid Syndromes: Two New Case Reports and Systematic Review” to “Life Expectancy and Causes of Death in Classical Laminopathic Progeroid Syndromes: Two New Case Reports and Systematic Review with Individual-Patient Data Synthesis” to better reflect the planned IPD synthesis. A typographical error in the number of records retrieved from Scopus (initially reported as 635) was corrected to 636, reflecting a counting mistake rather than a change in the study set. Funding and conflict-of-interest statements were updated to provide more detailed disclosure of external funding sources and of fees received by D.A.-V. from Amryt Pharmaceuticals and Regeneron Pharmaceuticals. These changes concern transparency and registration only and did not affect study selection, data extraction, risk-of-bias assessment or statistical methods.

No deviations affected the core eligibility criteria, main outcomes or the overall analytical strategy as registered in PROSPERO. Any implications of the design (case-report–based evidence, lack of formal reporting-bias and certainty assessments) for interpretation are explicitly discussed in the main manuscript.
