# Supplementary material for: Life expectancy and causes of death in classical laminopathic progeroid syndromes: systematic review with individual-patient data synthesis
Source: Aging (Albany NY). 2026 Jun 18;18(1):695–718. doi: 10.18632/aging.206389 (PMC13632853; doi:10.18632/aging.206389)
Supplement: Supplementary file 3 [file aging-18-1-206389-s007.pdf]

Coding rules:

1. **Cardiovascular Failure:**

   a. **Definition:** death primarily attributed to cardiac dysfunction, including heart failure, congestive heart failure, fatal arrhythmias, myocardial infarction, cardiovascular collapse, or sudden cardiac death of cardiac origin.

   b. **Coding rule:**

      i. Code as Cardiovascular Failure when the report explicitly identifies a cardiac cause of death, using terms such as “heart failure”, “cardiac failure”, “congestive heart failure”, “myocardial infarction”, “fatal arrhythmia”, “asystolic cardiac arrest”, “cardiovascular collapse”, or “sudden cardiac death”.

      ii. Alternatively, classify as Cardiovascular Failure only when (1) the report describes objective, severe, life-threatening cardiovascular pathology (e.g., advanced atherosclerosis, critical coronary stenosis, multivessel coronary disease, recurrent myocardial infarctions, or recent decompensated congestive heart failure), and (2) the patient dies suddenly or shortly after discharge without any competing non-cardiovascular cause mentioned. If these conditions are not met and no explicit cardiovascular cause is reported, do not classify the death as Cardiovascular Failure.

   c. **Exclusion rule:** do not code as Cardiovascular Failure when the report only states non-specific terms such as “cardiorespiratory arrest”, “respiratory and cardiac arrest”, without etiology. Code as Other in those cases.

2. **Ischemic stroke:**

   a. **Definition:** death resulting from clinically or radiologically confirmed ischemic cerebral infarction.

   b. **Coding rule:** code as Ischemic stroke when “ischemic stroke”, “cerebral infarction”, “brain ischemia”, or “cerebral arterial occlusion” is explicitly described as the cause of death.

   c. **Exclusion rule:** do not code as Ischemic Stroke when the report uses non-specific cerebrovascular terms such as “stroke”, “cerebrovascular accident”, or “cerebral event” without specifying that the event was ischemic.

3. **Trauma or accident:**

a. **Definition:** death due to non-medical traumatic events such as falls, injuries, domestic accidents, or traffic-related incidents.

b. **Coding rule:** code as Trauma when the report states: “trauma”, “fall”, “injury” or “accident-related death”.

c. **Excluding rule:** do not code as Trauma when:

    i. Perimortem injuries are described but are not identified as the cause of death

    ii. Deaths following trauma-related complications (e.g., sepsis, respiratory failure) should not be coded as Trauma unless the report explicitly states that the traumatic event itself was the primary cause of death.
    In such cases, classify according to the terminal mechanism

4. Respiratory failure:

a. **Definition:** death attributed to respiratory insufficiency including severe pneumonia, acute respiratory distress syndrome (ARDS), hypoventilation, or terminal respiratory failure.

b. **Coding rule:** code as Respiratory failure when:

    i. The report explicitly states a respiratory cause of death, using terms such as: “respiratory failure”, “respiratory arrest”, “fatal pneumonia”, “ARDS”, “respiratory insufficiency”, “pulmonary insufficiency” or “respiratory distress”.

    ii. The narrative indicates that death followed progressive, disease-related respiratory deterioration, even in the absence of specific diagnostic labels.

c. **Excluding rule:** do not code as Respiratory failure when the term “cardiorespiratory arrest” is mentioned without evidence that the primary event was respiratory. Code as Other in those cases.

5. Sepsis:

a. **Definition:** death caused by systemic infection, sepsis, or septic shock, regardless of the primary focus.

b. **Coding rule:** code as sepsis when: “sepsis”, “septic shock”, or “septicemia” is reported as the primary cause of death.

c. **Excluding rule:** do not code as sepsis when an alternative primary cause of death is clearly established, even if sepsis is mentioned as a secondary or contributing condition.

6. Other:

a. **Definition:** deaths that do not fit into any predefined category, including multifactorial deaths without a dominant system, non-specific terminal deterioration, isolated events, or postoperative complications not attributable to a single organ/system.

b. **Coding rule:** code as other when:

   i. The report clear mentions conditions or findings that do not fit any established category.

   ii. The death follows postoperative complications that are not clearly attributable to a specific organ/system.

   iii. The report describes isolated events (e.g., seizures/convulsions) as the cause of death, and no underlying condition can be classified within another category.

c. **Excluding rule:** do not code as other when the report clearly identifies a primary cause of death that fits another predefined category.

7. Unknown (n.a):

a. **Definition:** cause of death not reported or insufficiently described to assign a system-based category

b. **Coding rule:** code as unknown when:

   i. the report does not provide enough detail to identify the terminal event, mechanism, or dominant system responsible for death.

   ii. The cause of death is multifactorial, chronic, or related to long-term developmental or congenital abnormalities without a clear terminal mechanism.

c. **Excluding code:** do not code as unknown when:

i. The report contains any explicit system-based cause.

ii. The narrative provides enough detail to reasonably infer a primary system, even if several comorbidities coexist.

iii. Autopsy findings or clinical descriptions clearly point to a dominant terminal mechanism, even if not labeled as such in the text.
