# Supplementary material for: Life expectancy and causes of death in classical laminopathic progeroid syndromes: systematic review with individual-patient data synthesis
Source: Aging (Albany NY). 2026 Jun 18;18(1):695–718. doi: 10.18632/aging.206389 (PMC13632853; doi:10.18632/aging.206389)
Supplement: Supplementary file 4 [file aging-18-1-206389-s009.pdf]

SUPPLEMENTARY FIGURES

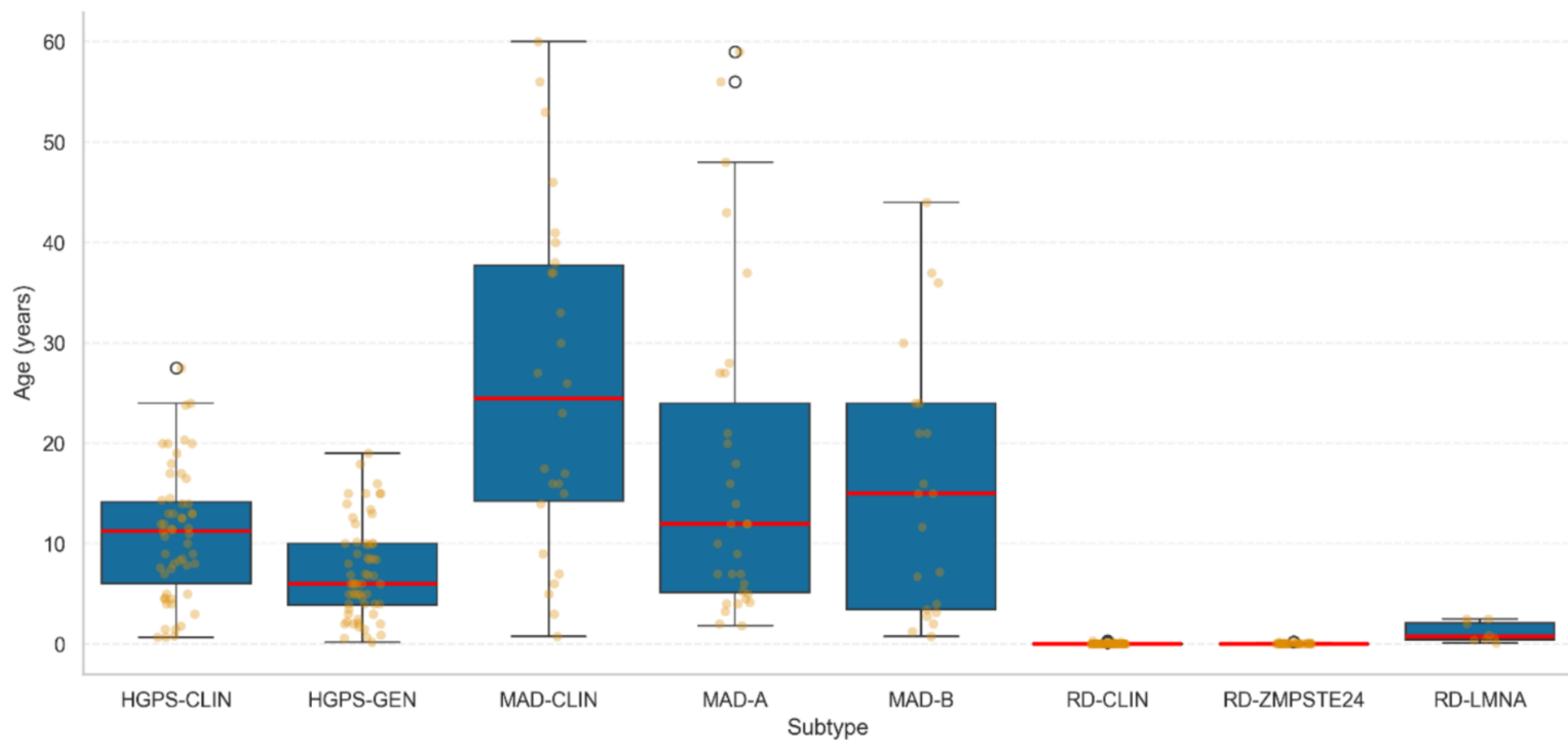

Supplementary Figure 1. Age distribution by subtype (all cases). Boxplots of age at last observation (years) by subtype including both clinical and genetically confirmed individuals. Boxes show the IQR, the red line is the median, whiskers extend to 1.5×IQR, and jittered dots represent individual participants (IPD). The subtypes follow pre-established definitions for genetically confirmed cases (HGPS, MAD-A, MAD-B, RD-*LMNA*, RD-*ZMPSTE24*) and cases with a clinical-only diagnosis (HGPS-CLIN, MAD-CLIN, RD-CLIN).

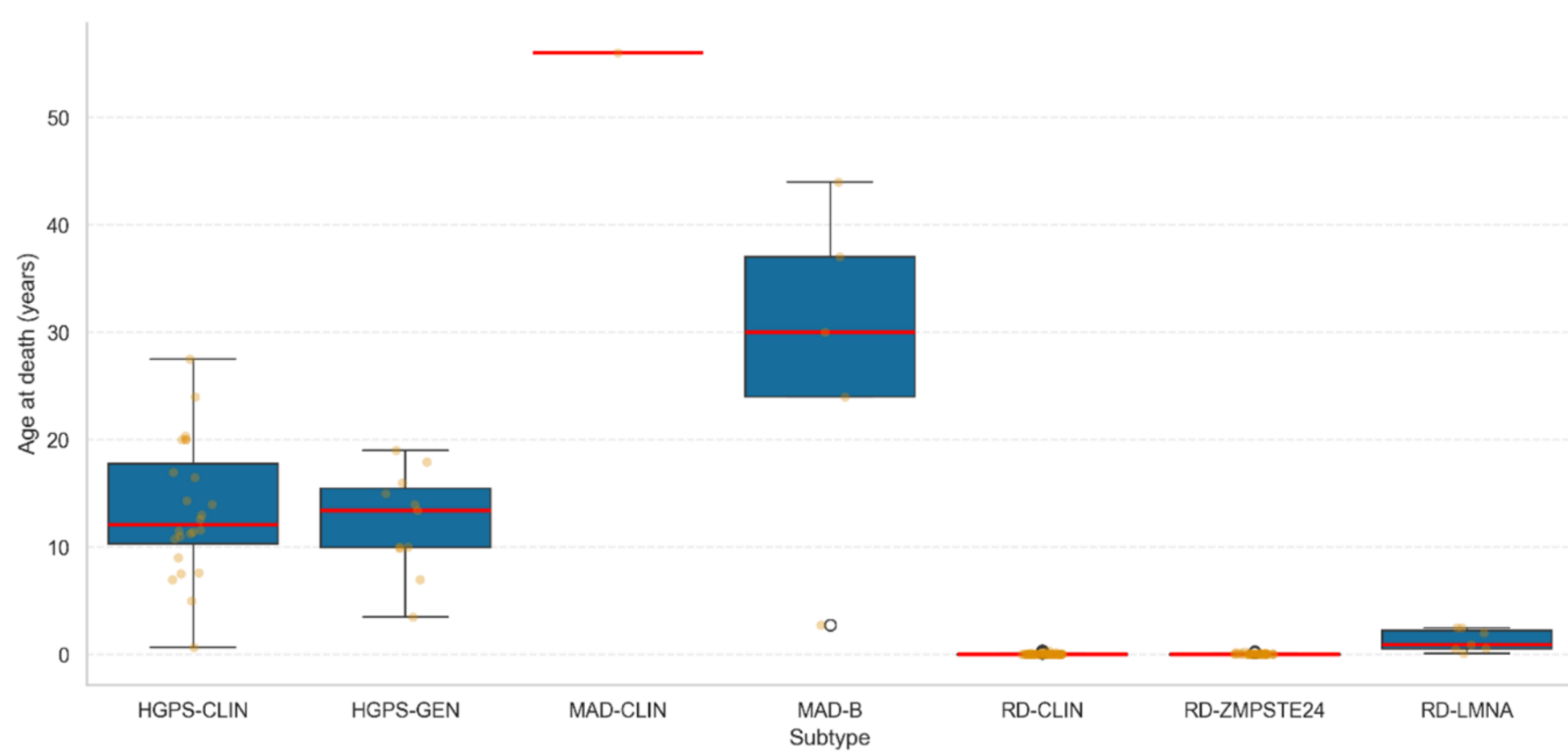

Supplementary Figure 2. Age at death by subtype (all cases). Boxplots of age at death (years) by subtype, restricted to participants with an observed death, including both clinical and genetically confirmed individuals. Boxes show the IQR, the red line is the median, whiskers = 1.5×IQR; points are individual deaths.

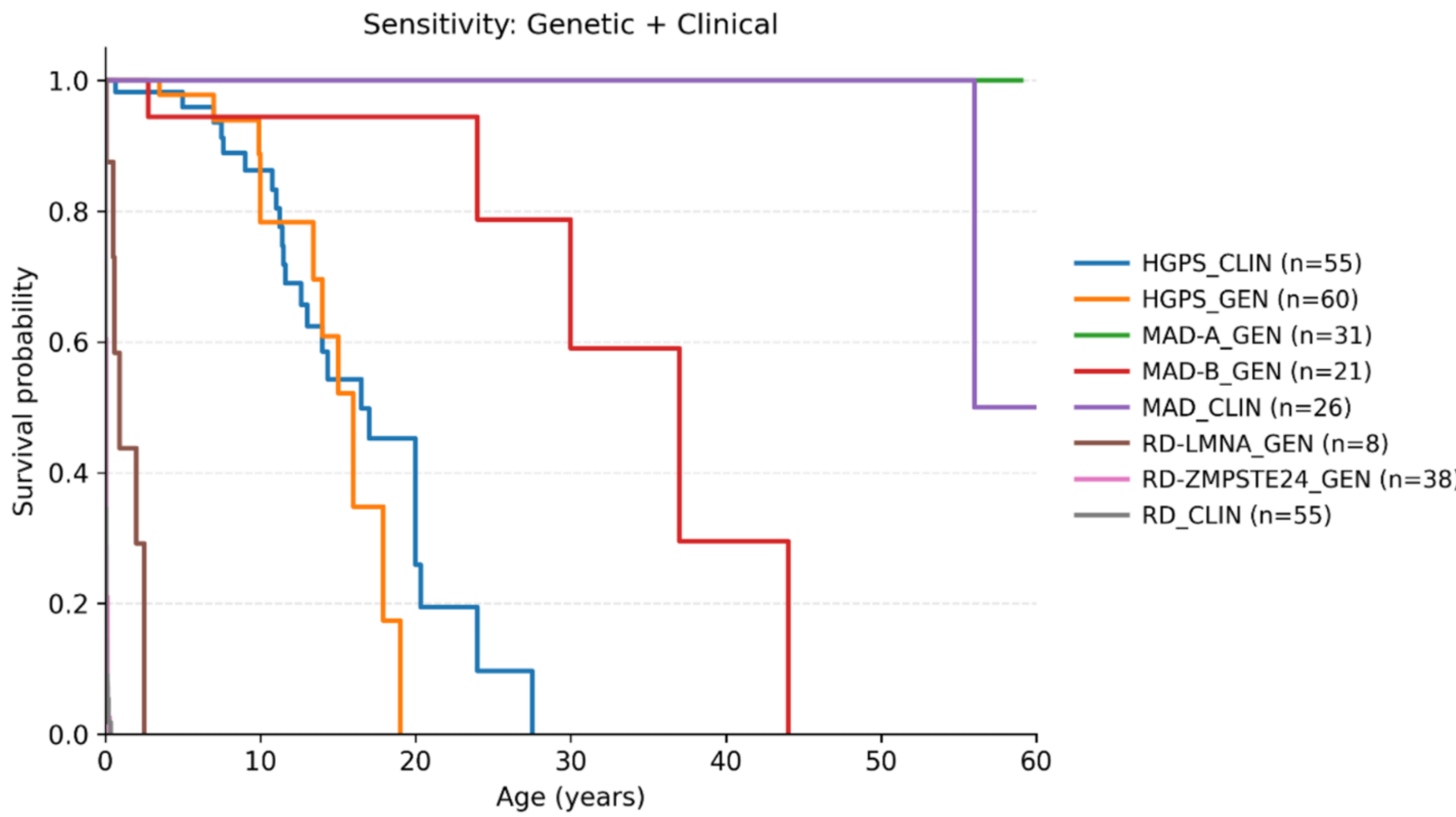

Supplementary Figure 3. Sensitivity analysis in Kaplan-Meier. Kaplan–Meier curves by subtype when clinically diagnosed cases (HGPS_CLIN, MAD-CLIN, RD-CLIN) are pooled with genetically confirmed cases (HGPS_GEN; MAD-A_GEN; MAD-B_GEN; RD-*LMNA*_GEN; RD-*ZMPSTE24*_GEN). Time scale is age (years). Medians and pairwise log-rank tests with Holm adjustment are reported in Supplementary Tables. This sensitivity complements the primary KM restricted to genetically confirmed IPD (Figure 2).

A.

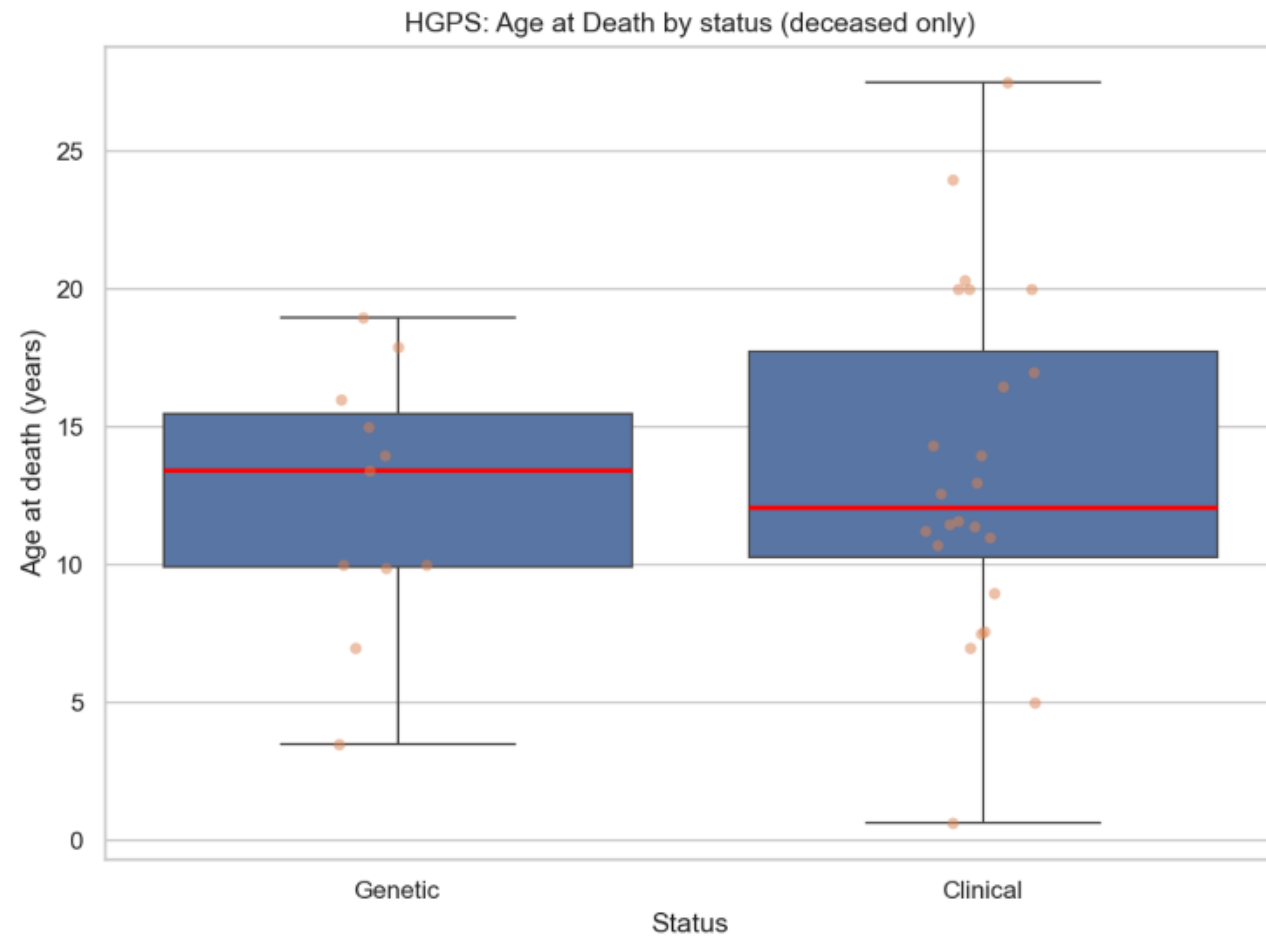

B.

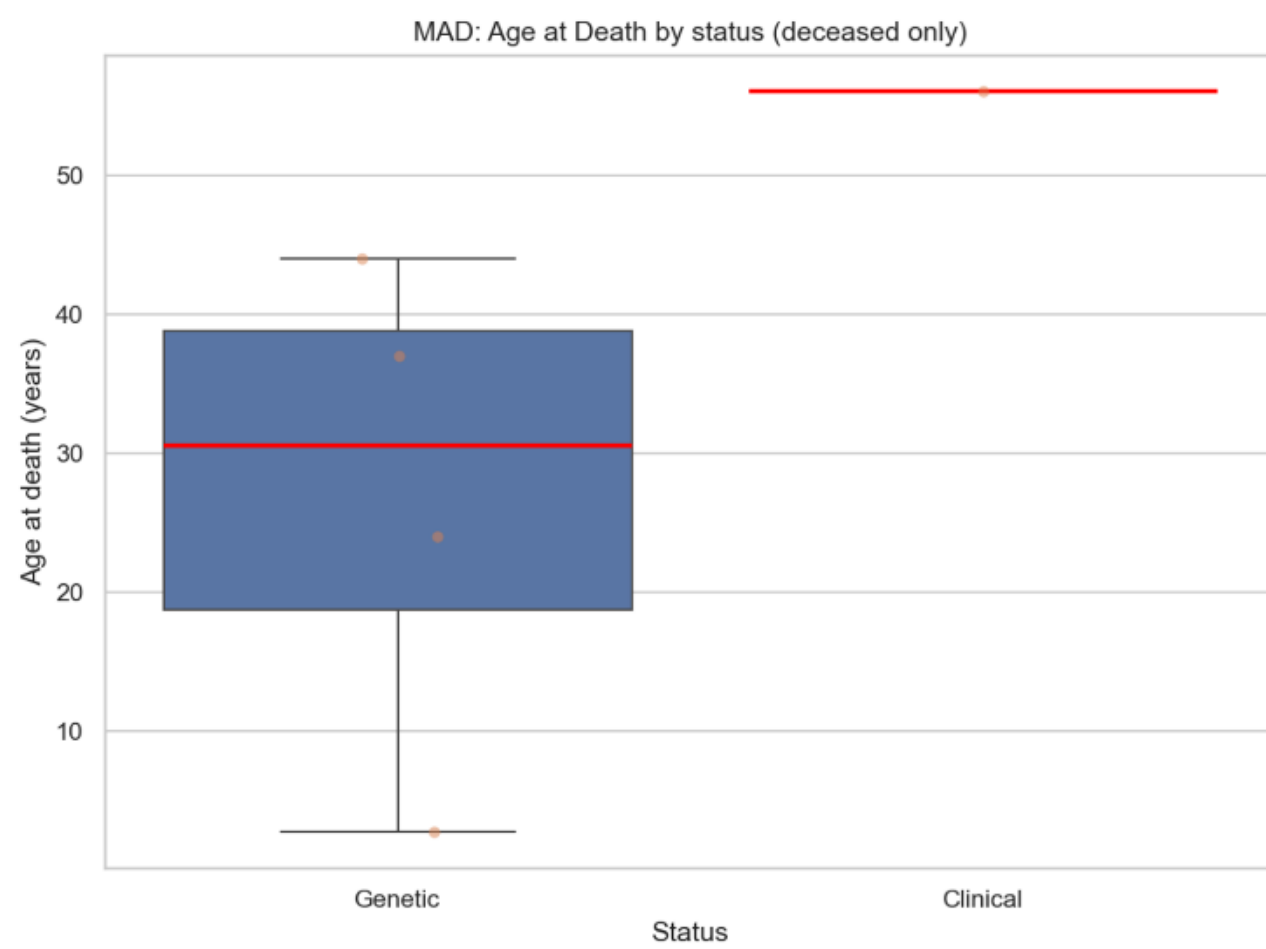

C.

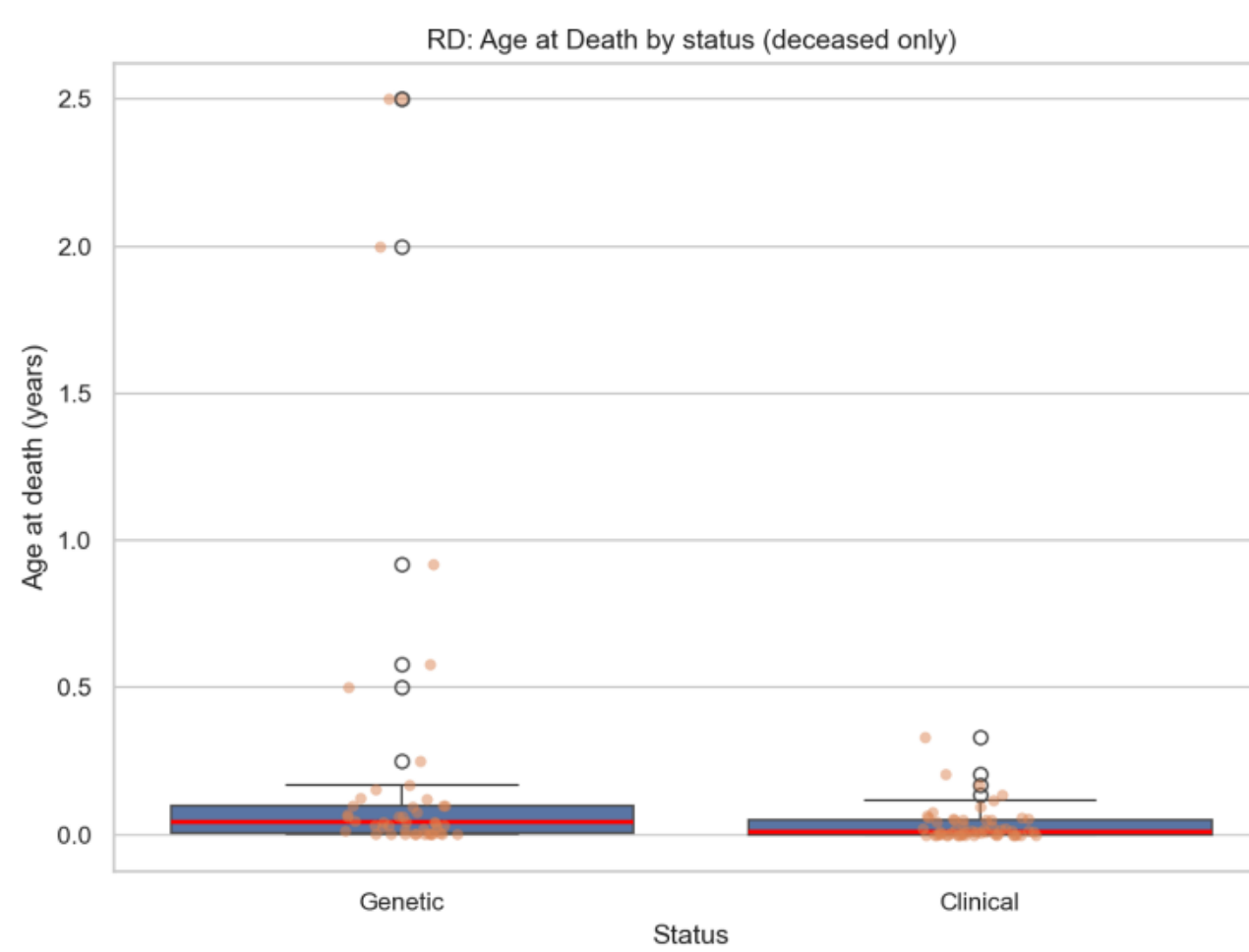

Supplementary Figure 4. (**A**–**C**) Age at death by diagnostic status within each disease (deceased only). (**A**) HGPS (clinical and genetic diagnoses), (**B**) MAD (clinical and genetical -A+B- diagnoses), (**C**) RD (clinical and genetical -*LMNA*+*ZMPSTE24*- diagnoses). Boxplots compare genetically confirmed vs. clinically diagnosed cases within each disease group. Boxes = IQR; red line = median; whiskers = 1.5×IQR; points = individual deaths.

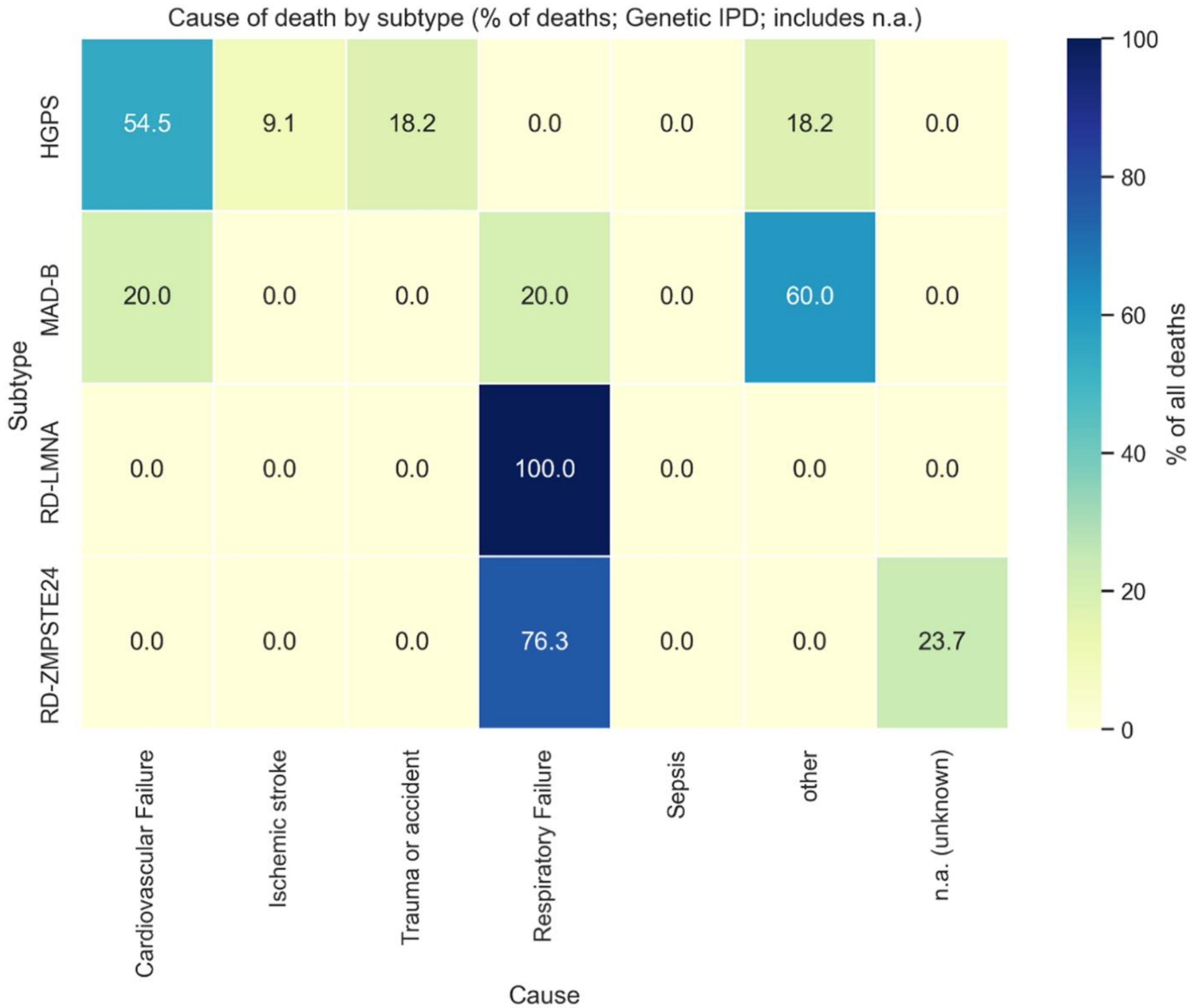

Supplementary Figure 5. Cause of death by subtype: heatmap (% of deaths; Genetic IPD). Heatmap showing row-wise percentages of mutually exclusive cause categories across subtypes in the primary cohort (genetically confirmed IPD). Cells are annotated with the percentage of all deaths within each subtype. Categories: cardiovascular failure, ischemic stroke, trauma/accident, respiratory failure, sepsis, other, and unknown (n.a.). Exact counts and 95% Wilson CIs (global) are provided in Supplementary Tables.

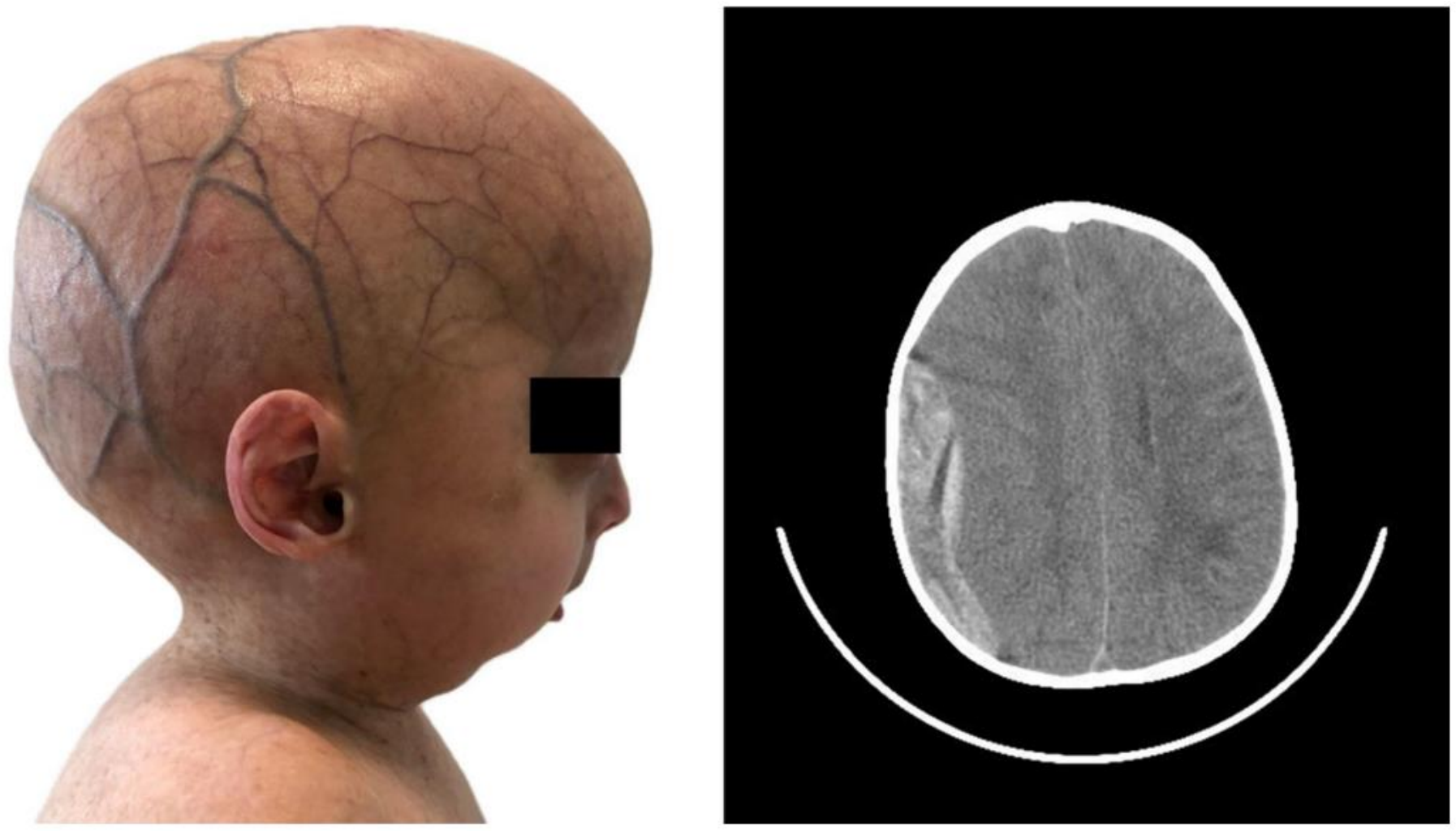

Supplementary Figure 6. Clinical phenotype and trauma-related intracranial haemorrhage in a child with classical HGPS (Case 1). Lateral clinical photograph of a 5-year-old boy with genetically confirmed classical Hutchinson–Gilford progeria syndrome (*LMNA* c.1822G>A, p.G608S), showing typical features including generalized alopecia, prominent cranial veins and micrognathia (left). A non-contrast axial head CT performed after traumatic brain injury demonstrates asymmetric post-traumatic intracranial haemorrhage (right). This figure illustrates the combination of severe physical frailty and susceptibility to trauma-related complications in HGPS.

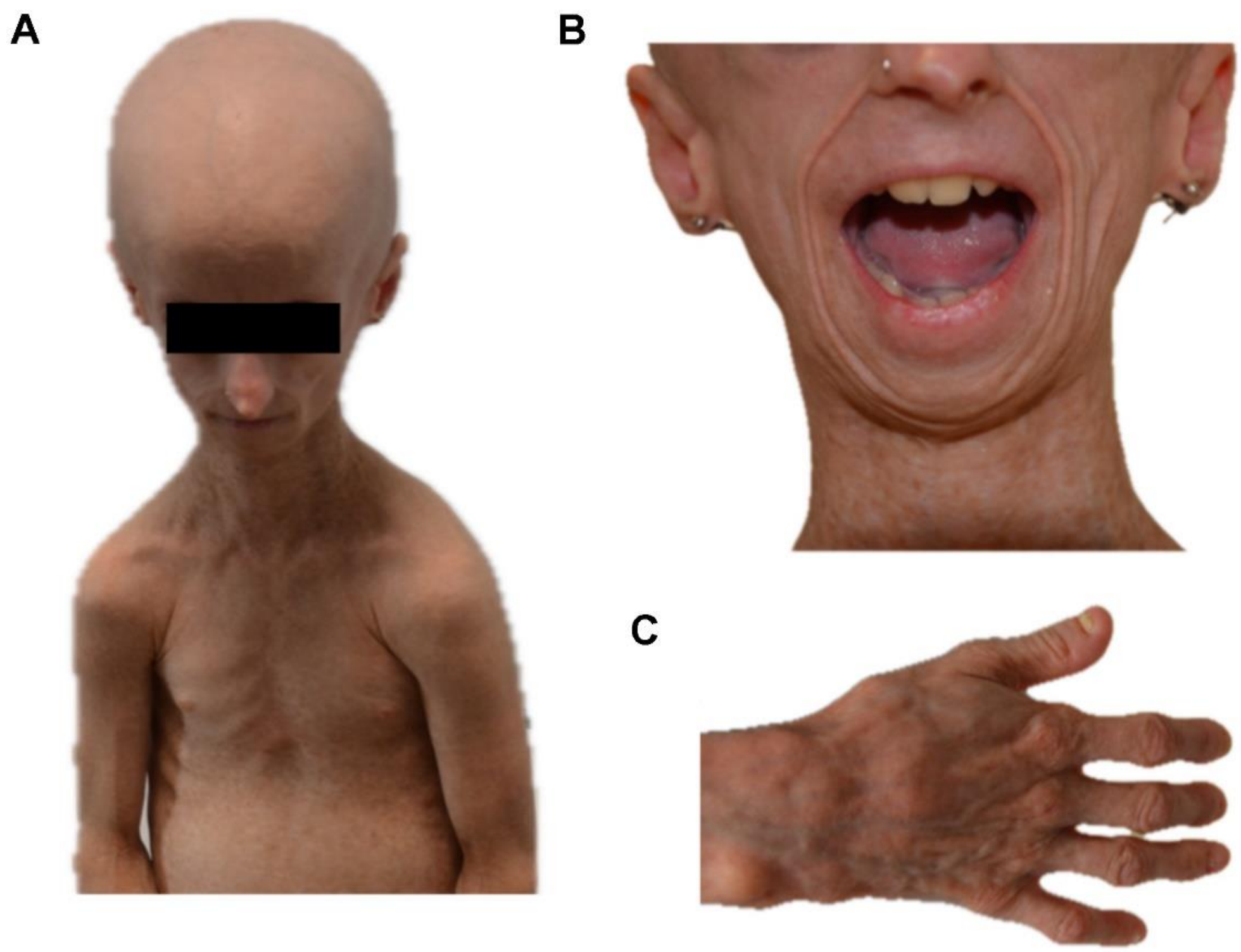

Supplementary Figure 7. Clinical phenotype of a 16-year-old girl with classical Hutchinson–Gilford progeria syndrome (Case 2). Clinical photographs of a 16-year-old girl with genetically confirmed classical Hutchin-son– Gilford progeria syndrome (*LMNA* c.1824C>T, p.G608G). (**A**) Frontal view showing characteristic craniofacial features, including alopecia, a pointed nose, micrognathia and sloping shoulders. (**B**) Open-mouth view illustrating dental abnormalities, reduced oral soft tissue volume and dental crowding. (**C**) Dorsal view of the hand demonstrating marked loss of subcutaneous fat, prominent veins, and sclerodermatous skin changes.

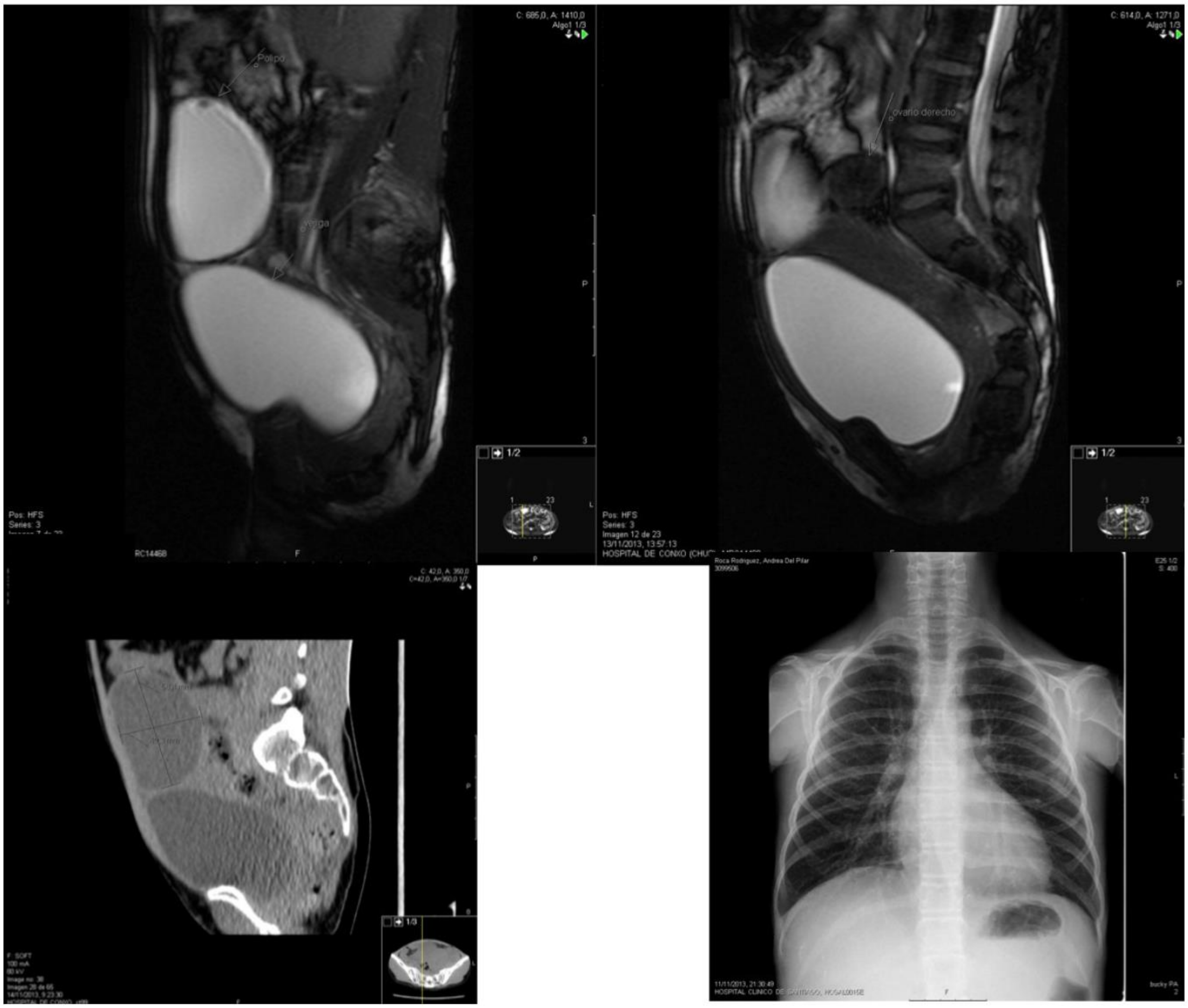

Supplementary Figure 8. Ovarian cyst and thoracic imaging in a 16-year-old girl with classical Hutchinson–Gilford progeria syndrome (Case 2). Pelvic and thoracic imaging in the 16-year-old girl with genetically confirmed classical Hutchinson–Gilford progeria syndrome (*LMNA* c.1824C>T, p.G608G) described in Case 2. The initial images demonstrate a large, well-defined, thin-walled right adnexal cyst with homogeneous fluid content, consistent with a simple ovarian cyst measuring approximately 8 × 3.5 cm and displacing adjacent pelvic structures (top panels). A posteroanterior chest radiograph shows no overt cardiopulmonary abnormalities (bottom right).

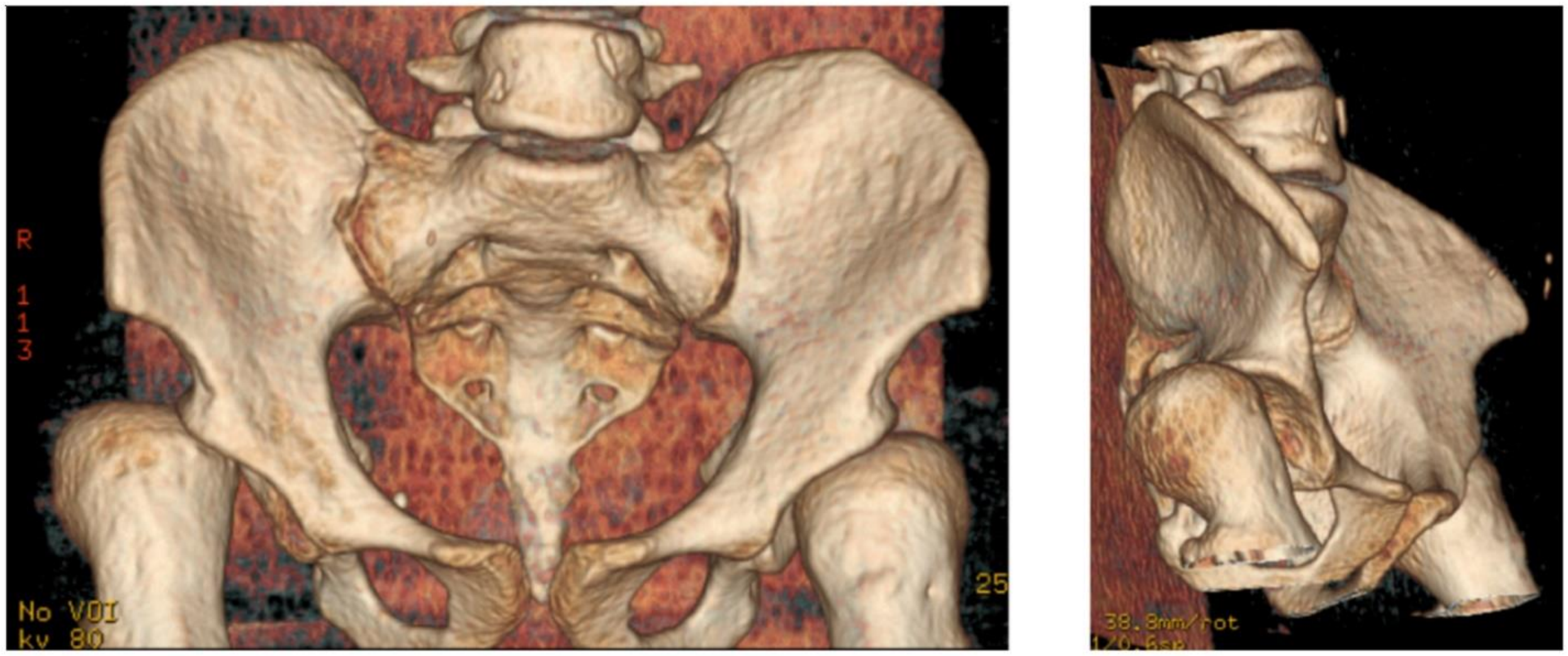

Supplementary Figure 9. Hip morphology in a 16-year-old girl with classical Hutchinson–Gilford progeria syndrome (Case 2). An anteroposterior pelvic radiograph of the 16-year-old girl with genetically confirmed classical Hutchinson–Gilford progeria syndrome (*LMNA* c.1824C>T, p.G608G) described in Case 2. The image shows bilateral coxa valga with shallow acetabula and gracile proximal femora, consistent with the reported hip dysplasia and joint stiffness.
