# Supplementary material for: Life expectancy and causes of death in classical laminopathic progeroid syndromes: systematic review with individual-patient data synthesis
Source: Aging (Albany NY). 2026 Jun 18;18(1):695–718. doi: 10.18632/aging.206389 (PMC13632853; doi:10.18632/aging.206389)
Supplement: Supplementary file 5 [file aging-18-1-206389-s010.pdf]

SUPPLEMENTARY TABLES

Supplementary Table 1. PICOS eligibility criteria.

**Population**	Patients of any age and sex with classic laminopathic progeria:<br>• Hutchinson-Gilford: LMNA c.1824C>T [p.G608G] and c.1822G>A [p.G608S])<br>• Mandibuloacral dysplasia type A: LMNA (p.R527H and others)<br>• Mandibuloacral dysplasia type B: ZMPSTE24<br>• Restrictive dermopathy: ZMPSTE24 or LMNA
**Intervention/ Exposure**	**Diagnosis of a classical laminopathic progeroid syndrome** (Hutchinson-Gilford progeria, mandibuloacral dysplasia type A/B, or restrictive dermopathy), established **either**<br>a) by pathogenic mutation in *LMNA* or *ZMPSTE24* confirmed by Sanger, targeted NGS panel, exome, or equivalent method, **or**<br>b) by clinical criteria clearly described by the authors
**Comparison**	1. Diagnostic method: genetically confirmed vs clinically diagnosed cases (within the same subtype when possible)<br>2. Between subtypes: HGPS vs MAD-A vs MAD-B vs RD
**Outcomes**	**Primary objectives**<br>1. Estimate pooled life expectancy in classical laminopathic progeroid syndromes.<br>2. Describe the overall distribution of causes of death (cardiovascular failure, ischemic stroke, trauma or accident, respiratory failure, sepsis, other and N.A. -unknown-).<br>**Secondary objectives**<br>3. Compare life expectancy and causes of death between genetically confirmed cases with clinically diagnosed cases.<br>4. Compare the same outcomes across sub-types.<br>5. Add two genetically confirmed cases from our centre to the pooled dataset.
**Design**	• Case reports and case series (≥1 patient) with extractable data: vital status, age at death or last known age (if alive), and cause of death (if deceased).<br>• Clinical / observational / comparative studies.<br>• Retrospective or prospective cohorts.

Supplementary Table 2. Cumulative incidence of cardiovascular and respiratory death at 1, 5, 10 and 20 years in classical laminopathic progeroid syndromes (competing-risks analysis).

Subtype	Time since birth (years)	CV death CIF (%)	RESP death CIF (%)
**HGPS**	1	0.0	0.0
**HGPS**	5	0.0	0.0
**HGPS**	10	4.8	0.0
**HGPS**	20	46.0	0.0
**RD-LMNA**	1	0.0	56.2
**RD-LMNA**	5	0.0	100.0
**RD-LMNA**	10	0.0	100.0
**RD-LMNA**	20	0.0	100.0
**RD-ZMPSTE24**	1	0.0	76.3
**RD-ZMPSTE24**	5	0.0	76.3
**RD-ZMPSTE24**	10	0.0	76.3
**RD-ZMPSTE24**	20	0.0	76.3

CV, cardiovascular death; RESP, respiratory death; CIF, cumulative incidence function. Estimates obtained from a competing-risks model. No Fine–Gray subdistribution hazard ratios are reported because of the limited number of cause-specific events in several strata.

Supplementary Table 3. Number of included patients and deaths by subtype in the primary and sensitivity analyses.
(A) Overall number of patients and deaths by scenario.

scenario	N_rows	N_unique_patients	Deaths
ALL_rows_input	294	294	141
Aggregated_raw	0	0	0
IPD_all	294	294	141
primary	158	158	61
S1_includingClinical	294	294	141
S2_noHighROB	154	154	57
S3_noOwn	156	156	59
S3_onlyOwn	2	2	2

(B) Distribution of patients by laminopathic subtype in each scenario.

scenario	HGPS_N	MAD-A_N	MAD-B_N	RD-LMNA_N	RD-ZMPSTE24_N	ATYPICAL_N	MAD-clin_N	RD-clin_N
ALL_rows_input	115	31	21	8	38	0	26	55
Aggregated_raw								
IPD_all	115	31	21	8	38	0	26	55
primary	60	31	21	8	38	0	0	0
S1_includingClinical	115	31	21	8	38	0	26	55
S2_noHighROB	60	31	21	8	34	0	0	0
S3_noOwn	58	31	21	8	38	0	0	0
S3_onlyOwn	2	0	0	0	0	0	0	0

Primary = genetically confirmed individual patient data (IPD) only.
S1 (*including clinical diagnoses*) = primary IPD plus patients diagnosed on clinical/phenotypic grounds without molecular confirmation.
S2 (*no high risk of bias*) = primary IPD excluding studies judged at high risk of bias in the IPD risk-of-bias assessment.
S3 (*no own centre*) = primary IPD excluding cases from our own centre; S3-onlyOwn = IPD from our own centre only (exploratory).
Full definitions of each scenario are provided in Supplementary File 1.

Supplementary Table 4. Pairwise log-rank and Tarone–Ware tests between laminopathic progeroid subtypes (genetically confirmed IPD cohort).

Subtype_A	Subtype_B	p_raw	p_tarone_ware	p_Holm
HGPS	MAD-A	9,5E-06	0,000148	2,85E-05
HGPS	MAD-B	0,000848	0,017876	0,001695
HGPS	RD-LMNA	1,5E-22	1,08E-21	1,35E-21
HGPS	RD-ZMPSTE24	4,34E-31	5,97E-29	4,34E-30
MAD-A	MAD-B	0,00243	0,008654	0,00243
MAD-A	RD-LMNA	3,76E-13	9,39E-13	2,26E-12
MAD-A	RD-ZMPSTE24	2,13E-18	1,43E-16	1,7E-17
MAD-B	RD-LMNA	2,82E-09	7,24E-09	1,41E-08
MAD-B	RD-ZMPSTE24	9,56E-14	4E-12	6,7E-13
RD-LMNA	RD-ZMPSTE24	3,23E-06	2,39E-05	1,29E-05

Pairwise comparisons of overall survival between genetically confirmed IPD subtypes using Kaplan–Meier curves.
p_raw: two-sided log-rank p-value.
p_Tarone–Ware: two-sided Tarone–Ware test p-value.
p_Holm: Holm–Bonferroni–adjusted p-value for 10 pairwise comparisons.
